# Protection against COVID-19 hospitalisation conferred by primary-series vaccination with AZD1222 in non-boosted individuals: first vaccine effectiveness results of the European COVIDRIVE study and meta-regression analysis

**DOI:** 10.1016/j.lanepe.2023.100675

**Published:** 2023-07-01

**Authors:** Wilhelmine Meeraus, Leonie de Munter, Christen M. Gray, Akshat Dwivedi, Chloé Wyndham-Thomas, Mario Ouwens, Wendy Hartig-Merkel, Laura Drikite, Griet Rebry, Antonio Carmona, Anke L. Stuurman, Thi Yen Chi Nguyen, Guillermo Mena, Ainara Mira-Iglesias, Giancarlo Icardi, Susana Otero-Romero, Sebastian Baumgartner, Charlotte Martin, Sylvia Taylor, Kaatje Bollaerts

**Affiliations:** a Medical Evidence, Vaccines & Immune Therapies, AstraZeneca, Cambridge, UK; b P95 Pharmacovigilance and Epidemiology, Leuven, Belgium; c Real World Science, BioPharmaceuticals Medical, AstraZeneca, Cambridge, UK; d Medical and Payor Statistics, BioPharmaceutical Business Unit, AstraZeneca, Mölndal, Sweden; e Vaccine Research Department, Fundación para el Fomento de la Investigación Sanitaria y Biomédica (FISABIO) de la Comunitat Valenciana, Salud Pública, Valencia, Spain; f Biomedical Research Consortium of Epidemiology and Public Health (CIBER-ESP), Instituto de Salud Carlos III, Madrid, Spain; g Preventive Medicine Department - Germans Trias i Pujol University Hospital, Badalona, Spain; h Autonomous University of Barcelona, Bellaterra, Spain; i Germans Trias I Pujol Research Institute (IGTP), Badalona, Spain; j Interuniversity Research Centre on Influenza and Other Transmissible Infections (CIRI-IT), Genoa, Italy; k Department of Health Sciences, University of Genoa, Italy; l Servicio de Medicina Preventiva y Epidemiología, Hospital Universitari Vall d'Hebron, Vall d'Hebron Barcelona Campus Hospitalari, Barcelona, Spain; m Fourth Medical Department with Infectious Diseases and Tropical Medicine, Klinik Favoriten/Kaiser-Franz-Josef Hospital, Vienna, Austria; n Infectious Diseases Department, Centre Hospitalier Universitaire Saint-Pierre, Brussels, Belgium

**Keywords:** AZD1222, COVID-19, COVIDRIVE, Durability, European, Hospitalisation, Meta-regression, Vaccine effectiveness

## Abstract

**Background:**

Vaccine effectiveness (VE) studies with long-term follow-up are needed to understand durability of protection against severe COVID-19 outcomes conferred by primary-series vaccination in individuals not receiving boosters. COVIDRIVE is a European public-private partnership evaluating brand-specific vaccine effectiveness (VE). We report a prespecified interim analysis of primary-series AZD1222 (ChAdOx1 nCoV-19) VE.

**Methods:**

Seven Study Contributors in Europe collected data on individuals aged ≥18 years who were hospitalised with severe acute respiratory infection (June 1st, 2021–September 5th, 2022) and eligible for COVID-19 vaccination prior to hospitalisation. In this test-negative case–control study, individuals were defined as test-positive cases or test-negative controls (SARS-CoV-2 RT-PCR) and were either fully vaccinated (two AZD1222 doses, 4–12 weeks apart, completed ≥14 days prior to symptom onset; no booster doses) or unvaccinated (no COVID-19 vaccine prior to hospitalisation). The primary objective was to estimate AZD1222 VE against COVID-19 hospitalisation. A literature review and meta-regression were conducted to contextualise findings on durability of protection.

**Findings:**

761 individuals were included during the 15-month analysis period. Overall AZD1222 VE estimate was 72.8% (95% CI, 53.4–84.1). VE was 93.8% (48.6–99.3) in participants who received second AZD1222 doses ≤8 weeks prior to hospitalisation, with spline-based VE estimates demonstrating protection (VE ≥ 50%) 30 weeks post-second dose. Meta-regression analysis (data from seven publications) showed consistent results, with ≥80% protection against COVID-19 hospitalisation through ∼43 weeks post-second dose, with some degree of waning.

**Interpretation:**

Primary-series AZD1222 vaccination confers protection against COVID-19 hospitalisation with enduring levels of VE through ≥6 months.

**Funding:**

10.13039/100004325AstraZeneca.

**Research in context**

**Evidence before this study**

Prior to this study, there was limited evidence on the durability of protection conferred by two-dose primary-series AZD1222 vaccination among individuals who do not or cannot receive booster vaccination. In Europe, this population comprises a substantial number of individuals, with approximately 20% of adults who received primary-series vaccination not having received a booster. The COVIDRIVE test-negative case–control (TNCC) study estimates vaccine effectiveness (VE) against COVID-19 hospitalisation in this population.

**Added value of this study**

To our knowledge, this is the first real-world European study to estimate the long-term VE against COVID-19 hospitalisation of primary-series AZD1222 during a period when Delta followed by Omicron were the predominant variants. Our overall confounder-adjusted VE estimate of 72.8% from this prespecified interim analysis suggests that individuals who did not receive booster dosing retained a level of protection against hospitalisation due to their primary-series vaccination (potentially beyond 30 weeks), indicating its potential ongoing contribution to preventing severe COVID-19 outcomes over the course of the pandemic. Our meta-regression provides findings supportive of VE estimates by time since vaccination.

**Implications of all the available evidence**

These findings support the ongoing value of AZD1222 in the global response to the pandemic, particularly in countries where vaccine coverage is lower and there may be challenges with rapidly implementing mass vaccination programmes. These interim analysis data on protection conferred by primary-series AZD1222 in the absence of subsequent booster dosing will be of relevance to countries in which booster programmes are not yet available.

## Introduction

Multiple vaccines were developed under accelerated timelines during the COVID-19 pandemic to protect against severe acute respiratory syndrome coronavirus 2 (SARS-CoV-2) infection and severe outcomes, saving an estimated 19.8 million lives during the first year of vaccination.^1^ Among the earliest available in the European Union (EU) were BNT162b2 (Pfizer), mRNA-1273 (Moderna), Ad26.COV2.S (Janssen) and AZD1222 (ChAdOx1 nCoV-19; AstraZeneca), with a total of six COVID-19 vaccines authorised for primary-series vaccination in the EU as of October 2022. Additional vaccines are licensed as boosters, including those offering protection against ancestral SARS-CoV-2 and newer variants.^2^ These vaccines continue to be critical in SARS-CoV-2 infection prevention and control at the population level, and offer protection to individuals against hospitalisation and death caused by COVID-19.^1^

In Europe, following a high uptake of primary-series COVID-19 vaccination (82.4% of the adult population [age ≥18 years] in 30 EU/European Economic Area [EEA] countries), there has been a reduced uptake of booster doses (first booster: 65.4% of the adult population; 79.3% of those receiving a primary series),^3^ highlighting the importance of understanding the protection against severe COVID-19 outcomes conferred by primary-series vaccination for individuals who choose not to or are unable to receive booster vaccination (an estimated 63.1 million adults in 30 EU/EEA countries^3^). Such data are also relevant for regions in which boosters are not yet available (49 countries primarily in central Africa, the Middle East and Asia^4^). Furthermore, understanding the durability of protection from primary-series vaccination in the context of currently circulating SARS-CoV-2 variants is of importance.

Most COVID-19 vaccines in the EU were initially granted conditional marketing authorisation, with the expectation that marketing authorisation holders (MAHs) would provide ongoing monitoring of vaccine effectiveness (VE) in real-world conditions. Real-world studies can provide important data with long-term follow-up on durability of protection against different outcomes and on waning effectiveness, data that are complementary to efficacy findings from clinical trials with typically shorter durations of follow-up. Such real-world effectiveness data can also provide a better understanding of how primary-series vaccination has contributed to the prevention of severe outcomes in Europe over the course of the pandemic.

COVIDRIVE is a public–private partnership that was established in November 2020 to address COVID-19 VE monitoring in the EU (https://covidrive.eu), enabling MAHs to provide brand-specific evidence of real-world effectiveness to regulators, including the European Medicines Agency (EMA). COVIDRIVE includes nine vaccine manufacturers (MAHs) and three public health institutes and research organisations. It was built on the existing DRIVE network, an Innovative Medicines Initiative project monitoring influenza VE in the EU (www.drive-eu.org). COVIDRIVE uses harmonised methodology and operations, with a consistent master protocol (D8111R00005), to estimate brand-specific VE for participating MAHs.

AstraZeneca, which developed and manufactured AZD1222 in a non-profit programme in partnership with Oxford University, is a partner in the COVIDRIVE consortium and the first MAH to evaluate VE using the COVIDRIVE study platform. AZD1222, a replication-deficient simian adenovirus-vectored vaccine,^5^ is the most widely distributed vaccine worldwide, with 3 billion doses provided, and saved an estimated 6.3 million lives globally in the first year of vaccination.^1^^,^^6^ In its pivotal Phase III trial (NCT04516746),^7^ AZD1222 demonstrated durable protection against primarily ancestral SARS-CoV-2, plus some of the earlier variants, through a median follow-up time of 6 months, with an efficacy against severe/critical disease of 92.1%. The EMA granted conditional EU marketing authorisation for AZD1222 on January 29th, 2021, and full marketing authorisation for use as primary-series vaccination or as a heterologous or homologous booster on October 31st, 2022.^8^ The primary-series vaccination consists of two doses administered 4–12 weeks apart.^8^

Real-world evidence demonstrates the effectiveness of two-dose primary-series AZD1222 against COVID-19, with ≥80% effectiveness against hospitalisation due to SARS-CoV-2 variants including Alpha, Delta, and Omicron.^9^, ^10^, ^11^ Durability of AZD1222 effectiveness has been demonstrated in real-world evidence from the UK and Canada showing protection remaining high at 4–6 months against hospitalisation and death.^9^^,^^12^ However, longer-term durability of protection and protection against the more recently circulating Omicron subvariants are required to inform ongoing global use of primary-series AZD1222 and the need for subsequent booster dosing. Here we report VE and durability data from a prespecified interim analysis of the COVIDRIVE AZD1222-specific study (D8111R00017), the first results reported from the COVIDRIVE study platform, together with a literature review and meta-regression to contextualise our findings and to evaluate durability of effectiveness of primary-series AZD1222 across multiple studies.

## Methods

### COVIDRIVE study design

The COVIDRIVE study is an ongoing non-interventional, prospective and retrospective, multi-country, multicentre, hospital-based case–control study with test-negative controls (test-negative case–control [TNCC] design). It is designed to estimate the effectiveness of COVID-19 vaccines against COVID-19 hospitalisation (i.e., laboratory-confirmed SARS-CoV-2 infection in individuals hospitalised with a severe acute respiratory infection [SARI]). The study is being conducted based on a master protocol in multiple European countries and is overseen by a quality assurance and audit committee, and an independent scientific committee to safeguard scientific excellence. The master protocol was approved by independent ethics committees (IECs) at each Study Contributor (participating study sites that are either individual hospitals or hospital networks) and is registered with the European Network of Centres for Pharmacoepidemiology and Pharmacovigilance (ENCePP, EUPAS42328). This publication reports the second prespecified interim analysis results of the COVIDRIVE AZD1222-specific study, incorporating data from seven Study Contributors representing 13 hospitals in four countries – Austria, Belgium, Italy and Spain. This interim analysis, which was conducted based on reaching the prespecified sample size and on regulatory commitments for timing of interim analyses of the study, covers hospitalisations due to SARI during the period June 1st, 2021, to September 5th, 2022. A final analysis will be conducted upon reaching the prespecified sample size for this analysis, estimated to occur at approximately 18 months from the start of prospective data collection.

### COVIDRIVE participants

The study includes individuals hospitalised for SARI during the study period at a participating Study Contributor hospital, with hospitalisation defined as admission with at least one overnight stay for a SARI (defined based on suspicion of a respiratory infection with at least one of the following symptoms—cough, fever, shortness of breath, sudden onset of anosmia, ageusia or dysgeusia—with symptom onset within the 14 days prior to hospital admission, per the case definition from the European Centre for Disease Prevention and Control [ECDC]).^13^ For inclusion in the study, individuals were required to have been eligible for COVID-19 vaccination per their national or regional immunisation recommendations prior to hospital admission; per its EU authorisation, the AZD1222-specific study includes individuals aged ≥18 years. Individuals were excluded if they had been hospitalised due to COVID-19 within 3 months prior to their current hospital admission (hospital transfers were not considered a prior hospitalisation), or if they could not be swabbed due to severe nasal septum deviation, nasal obstruction or other conditions that contraindicated nasopharyngeal swabbing.

Individuals (or their legally acceptable representative) provided informed consent when applicable. Waiver of informed consent was authorised by local IECs for prospective data collection for Germans Trias i Pujol and Vall d'Hebron as the tests carried out and information collected are part of routine clinical practice and epidemiological surveillance. Additionally, waiver of consent was approved for retrospective data for all sites participating in retrospective data collection.

The present analysis includes both ‘fully vaccinated’ and ‘unvaccinated’ participants. ‘Fully vaccinated’ participants were those who had received a complete primary series of AZD1222 (and no other COVID-19 vaccine) consisting of two doses administered 4–12 weeks apart, with the second dose received ≥14 days prior to SARI symptom onset. ‘Unvaccinated’ participants were those who had not received any COVID-19 vaccine prior to hospitalisation due to SARI.

### Data collection

Study participants were recruited retrospectively and prospectively. Seven Study Contributors were participating in COVIDRIVE at the time of this analysis, and these Contributors collected data prospectively from September 15th, 2021 (earliest site), to September 5th, 2022, with five also collecting data retrospectively from hospital records on individuals hospitalised with SARI between June 1st, 2021 (earliest site), to September 5th, 2022 (Supplementary Table S1). Data on COVID-19 vaccination status, date, dose and vaccine received were required for all participants. Mandatory covariates collected by all Study Contributors for all participants were age, sex, chronic conditions, pregnancy, body mass index, and influenza and pneumococcus vaccination history. Additional optional covariates that were collected by the Study Contributors included prior SARS-CoV-2 infection, smoking history, long-term care facility residence, and whether the individual was a healthcare worker. Sources for exposure ascertainment could include vaccination registries, medical records or vaccination cards. Multiple data sources, including hospital medical records, vaccine registries, general practitioner medical records and on-study data collection, were also used to collect additional variables of interest. Data were entered into an electronic Case Report Form (eCRF, Castor®), with only pseudonymised data being transferred from the Study Contributors to the COVIDRIVE research server, a dedicated, secured central server hosted by P95.

### Objectives and assessments

The primary objective is to estimate the VE of AZD1222 against hospitalisation due to laboratory-confirmed SARS-CoV-2 in SARI patients who have been vaccinated with two doses, with a secondary objective to estimate AZD1222 VE by time since last dose, and an exploratory objective to estimate AZD1222 VE by SARS-CoV-2 genetic variant.

The TNCC design estimates VE among study participants fully vaccinated with two doses of AZD1222 versus unvaccinated study participants. Use of this TNCC design, in which controls are symptomatic patients who test negative for the pathogen of interest (SARS-CoV-2), reduces confounding due to healthcare-seeking behaviour and is an efficient design that is well suited to studying rare outcomes.^14^ As well as its frequent use for estimating COVID-19 VE, the TNCC design is routinely used for evaluating VE of influenza and rotavirus vaccines.^15^

In COVIDRIVE, ‘test-positive cases’ are defined as eligible study participants who met the SARI case definition and who tested positive for SARS-CoV-2 on at least one reverse transcription polymerase chain reaction (RT-PCR) assay (or RNA amplification assay with the same sensitivity) of a sample collected between 14 days prior to (day -14) or on the day of (day 0, within 24 h upon arrival) hospital admission. ‘Test-negative controls᾿ are defined as eligible study participants who met the SARI case definition and who tested negative for SARS-CoV-2 on all RT-PCR or similar molecular assays of samples collected between day −14 and day 0 (negative result required within 24 h of admission). SARS-CoV-2-positive samples collected from participants who were prospectively recruited to COVIDRIVE were genetically sequenced either locally or by a national reference laboratory using commercially available molecular kits. The variants were identified according to Pangolin lineage. Sequencing was not possible for retrospectively recruited participants.

### Statistical methods

All analyses were performed in R v4.0.0 or higher.

Simulation-based sample size calculations were performed at the start of the study and were then updated during the study period with assumptions reflective of the collected data, which were: an expected overall primary-series vaccination coverage rate of 85%, an AZD1222-specific brand-share of 6% among vaccinated individuals, a case–control ratio of 3:1, a combined VE of 80% for all other COVID-19 vaccines, and an anticipated VE of 80% for AZD1222. This resulted in a target sample size of 229 COVID-19 cases required to obtain VE estimates with an expected 95% confidence interval (CI) range of ≤50%. To offset potential loss of precision due to covariate adjustment, this sample size estimate was multiplied by a factor of 1.2 to give a target sample size of 274 COVID-19 cases for the primary objective at the present interim analysis.

VE estimates were obtained by transforming the odds ratios (ORs), using VE = (1 – OR) x 100%, from additive fixed-effect logistic regression models using data pooled across Study Contributors and with Study Contributor as a fixed effect. ORs were calculated as the odds of having been fully vaccinated with AZD1222 among test-positive cases (COVID-19 hospitalisations) divided by the odds of having been fully vaccinated with AZD1222 among test-negative controls (hospitalisation due to non-COVID-19 SARI).

Two analyses were conducted adjusting for the following confounders: 1) symptom onset date, age, sex and number of chronic conditions, and 2) symptom onset date. Symptom onset date was included in the model to adjust for changes in SARS-CoV-2 SARI incidence during the study period, which had multiple modes. For each month, the cubic regression spline included a knot corresponding to the first day of the month. Coefficients of the logistic regression models were estimated using restricted maximum likelihood estimation (REML), which also selects an optimal smoothing parameter for the spline effects. Age effect was modelled with a cubic regression spline with knots corresponding to 50, 65 and 80 years of age. Each time, complete-case analyses were performed. VE estimates were obtained overall and, for the secondary and exploratory objectives, by time since last dose and by SARS-CoV-2 genetic variant.

### Literature review and meta-regression

In support of the findings reported herein from the COVIDRIVE study, a literature review and meta-regression were conducted to identify other studies describing the durability of AZD1222 effectiveness over time in the general population. Studies were identified from three sources: the ‘Duration of protection weekly summary table’ published by the International Vaccine Access Center on VIEW-hub,^4^ a ‘living’ (i.e. regularly updated) systematic review of VE studies (accessed April 1st, 2022, and September 22nd, 2022); a US case–control study report that summarised relevant literature^16^; and a systematic literature review.^17^ For inclusion in the analysis, studies had to report data on severe (i.e. hospitalised) COVID-19 for a general adult population (defined as aged ≥16 or ≥18 years) who had received a two-dose primary series of AZD1222; studies were excluded if VE was estimated in a non-general population (e.g. healthcare workers), measured for a single time-point, or measured from time of first dose rather than second dose. Preprints were allowed for articles not published in journals. Data were preferentially extracted from published articles if both preprint and published article were available. Studies were identified and data were extracted by one of two researchers (ALS or TYCN) and checked by an independent reviewer (WM, ALS or TYCN). Data were extracted on study type, population characteristics, fully adjusted VE estimates (including 95% CIs) from ≥14 days post-second vaccine dose, and time-period for which the VE estimates applied.

The time-period for each VE was imputed as the mid-point of each time range in terms of time since complete vaccination. Variance was derived from each of the published 95% CIs. Where no cases were observed and no 95% CI published, variance was derived using the ‘rule of 3’, whereby the upper 95% CI of a 0/n rate is approximately 3/n.^18^ Meta-regression was performed by inverse weighting by variances of the VE estimates published in each study with random effects at the study and sub-group level.

### Role of the funding source

AstraZeneca is a partner in the COVIDRIVE consortium and funded the AZD1222-specific study. AstraZeneca authors conducted the literature search and meta-regression. AstraZeneca funded medical writing assistance for the development of the manuscript under the direction of the authors.

## Results

### COVIDRIVE study population

Of the 4758 individuals hospitalised with SARI and enrolled in the COVIDRIVE study from June 1st, 2021, to September 5th, 2022, 942 were either unvaccinated against COVID-19 or had received vaccination with AZD1222 as their last dose and were thus evaluated for inclusion in this analysis. Of the 942, 94 did not meet the eligibility criteria and 87 were excluded (31 received only one dose of AZD1222, 42 had a dosing interval of <4 or >12 weeks and 14 were missing data for analysis). A total of 761 individuals were thus included in the analysis; 561 were test-positive cases and 200 were test-negative controls (Supplementary Figure S1).

Participant characteristics are summarised in Table 1, with stratification by country in Supplementary Table S2. The median age was 60.0 years, and 37.8% of participants were aged ≥65 years. Of 397 participants with available information, 135 (34.0%) were obese. Overall, 442 (58.1%) participants had ≥1 chronic condition, with 303 (39.8%) having ≥2 such conditions; the most common were hypertension (33.1%), cardiovascular disease (21.8%), lung disease (18.1%) and type 2 diabetes (17.5%), and 7.0% of participants had an immunodeficiency. Analysis of associations between covariates and test status (*p* ≤ 0.05) showed that test-negative controls were somewhat older than the test-positive cases (46.5% vs 34.8% aged ≥65 years), were more frequently current (27.5% vs 8.2%) or previous (24.5% vs 18.0%) smokers, and had higher rates of chronic conditions including higher rates of cardiovascular disease (32.5% vs 18.4%), lung disease (32.5% vs 13.4%), cancer (17.0% vs 6.6%), chronic kidney disease (13.0% vs 7.5%), asthma (11.0% vs 4.6%) and immunodeficiency (12.0% vs 5.2%); they were also more commonly vaccinated for influenza (17.5% vs 6.6%) and pneumococcus (19.5% vs 4.8%) (Table 1). Among female participants, pregnancy was more common among test-positive cases than test-negative controls (8.6% vs 1.3%).

**Table 1:** Demographic characteristics of the study population.

	Total (N = 761)	Test-negative controls (n = 200)	Test-positive cases (n = 561)
**Age at hospitalisation, years**	60.0 (44.0–69.0)	63.0 (49.0–69.2)	58.0 (43.0–69.0)
18–49	248 (32.6)	53 (26.5)	195 (34.8)
50–64	225 (29.6)	54 (27.0)	171 (30.5)
≥65	288 (37.8)	93 (46.5)	195 (34.8)
**Sex**			
Female	323 (42.4)	78 (39.0)	245 (43.7)
Male	438 (57.6)	122 (61.0)	316 (56.3)
**Country/Study contributor**^a^			
Austria/Klinik Favoriten	79 (10.4)	2 (1.0)	77 (13.7)
Belgium	153 (20.1)	24 (12.0)	129 (23.0)
St Pierre	25 (3.3)	11 (5.5)	14 (2.5)
Universitair Ziekenhuis Antwerpen	128 (16.8)	13 (6.5)	115 (20.5)
Italy/CIRI-IT	131 (17.2)	36 (18.0)	95 (16.9)
Spain	398 (52.3)	138 (69.0)	260 (46.3)
FISABIO	127 (16.7)	43 (21.5)	84 (15.0)
Hospital Germans Trias i Pujol	171 (22.5)	75 (37.5)	96 (17.1)
Hospital Universitari Vall d'Hebron	100 (13.1)	20 (10.0)	80 (14.3)
**Body mass index (BMI) category**			
Underweight (BMI <18.5)	6 (0.8)	1 (0.5)	5 (0.8)
Normal/healthy weight (BMI ≥18.5 to <25.0)	124 (16.3)	44 (22.0)	80 (14.3)
Overweight (BMI ≥25.0 to <30.0)	132 (17.3)	33 (16.5)	99 (17.6)
Obese (BMI ≥30.0)	128 (16.8)	24 (12.0)	104 (18.5)
No information	7 (0.9)	1 (0.5)	6 (1.1)
Missing	364 (47.8)	97 (48.5)	267 (47.6)
**Number of chronic conditions**			
0	319 (41.9)	54 (27.0)	265 (47.2)
1	156 (20.5)	44 (22.0)	112 (20.0)
2	139 (18.3)	46 (23.0)	93 (16.6)
≥3	147 (19.3)	56 (28.0)	91 (16.2)
Excluding immunodeficiency	n = 708	n = 176	n = 532
0	319 (45.1)	54 (30.7)	265 (49.8)
1	147 (20.8)	41 (23.3)	106 (19.9)
2	118 (16.7)	37 (21.0)	81 (15.2)
≥3	124 (17.5)	44 (25.0)	80 (15.0)
**Participants with chronic condition**^b^			
Asthma	48 (6.3)	22 (11.0)	26 (4.6)
Lung disease	138 (18.1)	63 (32.5)	75 (13.4)
Cardiovascular disease	166 (21.8)	63 (32.5)	103 (18.4)
Hypertension	252 (33.1)	69 (34.5)	183 (32.6)
Chronic liver disease	32 (4.2)	9 (4.5)	23 (4.1)
Chronic kidney disease	68 (8.9)	26 (13.0)	42 (7.5)
Type 2 diabetes	133 (17.5)	29 (14.5)	104 (18.5)
Cancer	71 (9.3)	34 (17.0)	37 (6.6)
Immunodeficiency (or organ transplant)	53 (7.0)	24 (12.0)	29 (5.2)
**Pregnancy**	n = 323	n = 78	n = 245
No	288 (89.2)	76 (97.4)	212 (86.5)
Yes	22 (6.8)	1 (1.3)	21 (8.6)
No information	10 (3.1)	1 (1.3)	9 (3.7)
Missing	3 (0.9)	0	3 (1.2)
**Prior SARS-CoV-2 infection**^c^			
No prior infection recorded	503 (66.1)	120 (60.0)	383 (68.3)
Yes – clinical diagnosis	3 (0.4)	1 (0.5)	2 (0.4)
Yes – laboratory-confirmed	19 (2.5)	13 (6.5)	6 (1.1)
No information	2 (0.3)	0	2 (0.4)
Missing	234 (30.7)	66 (33.0)	168 (29.9)
**Vaccinated for influenza within 12 months prior to SARI hospital admission**			
No	485 (63.7)	144 (72.0)	341 (60.8)
Yes	72 (9.5)	35 (17.5)	37 (6.6)
No information	185 (24.3)	19 (9.5)	166 (29.6)
Missing	19 (2.5)	2 (1.0)	17 (3.0)
**Received any pneumococcal vaccine**			
No	483 (63.5)	139 (69.5)	344 (61.3)
Yes	66 (8.7)	39 (19.5)	27 (4.8)
No information	194 (25.5)	20 (10.0)	174 (31.0)
Missing	18 (2.4)	2 (1.0)	16 (2.9)
**Smoking history**			
Never smoked	258 (33.9)	47 (23.5)	211 (37.6)
Ex-smoker	150 (19.7)	49 (24.5)	101 (18.0)
Occasional smoker	13 (1.7)	7 (3.5)	6 (1.1)
Daily smoker	88 (11.6)	48 (24.0)	40 (7.1)
No information	243 (31.9)	45 (22.5)	198 (35.3)
Missing	9 (1.2)	4 (2.0)	5 (0.9)
**Long-term care facility resident**			
No	737 (96.8)	190 (95.0)	547 (97.5)
Yes	4 (0.5)	1 (0.5)	3 (0.5)
No information	11 (1.4)	5 (2.5)	6 (1.1)
Missing	9 (1.2)	4 (2.0)	5 (0.9)
**Healthcare worker**			
No	583 (76.6)	148 (74.0)	435 (77.5)
Yes	2 (0.3)	0	2 (0.4)
No information	167 (21.9)	48 (24.0)	119 (21.2)
Missing	9 (1.2)	4 (2.0)	5 (0.9)
**Period of disease onset**			
May–Jun 2021^d^	55 (7.2)	5 (2.5)	50 (8.9)
Jul–Sep 2021	142 (18.7)	32 (16.0)	110 (19.6)
Oct–Dec 2021	307 (40.3)	83 (41.5)	224 (39.9)
Jan–Mar 2022	188 (24.7)	51 (25.5)	137 (24.4)
Apr–Jun 2022	55 (7.2)	24 (12.0)	31 (5.5)
Jul–Aug 2022	14 (1.8)	5 (2.5)	9 (1.6)
**Genetic variant**^e^			
Alpha	1	NA	1 (0.2)
Beta	1	NA	1 (0.2)
Delta	123	NA	123 (21.9)
Omicron	86	NA	86 (15.3)
Not interpretable/unknown	2	NA	2 (0.4)
Missing	348	NA	348 (62.0)
**Vaccinated against COVID-19**			
No	641 (84.2)	140 (70.0)	501 (89.3)
Fully vaccinated with two doses of AZD1222	120 (15.8)	60 (30.0)	60 (10.7)
**Month and year of second AZD1222 dose**^f^	n = 120	n = 60	n = 60
March 2021	1 (0.8)	0	1 (1.7)
April 2021	0	0	0
May 2021	5 (4.2)	3 (5.0)	2 (3.3)
June 2021	33 (27.5)	19 (31.7)	14 (23.3)
July 2021	75 (62.5)	34 (56.7)	41 (68.3)
August 2021	4 (3.3)	3 (5.0)	1 (1.7)
September 2021	2 (1.7)	1 (1.7)	1 (1.7)

Data shown are n (%) or median (IQR).

CIRI-IT = Interuniversity Research Centre on Influenza and Other Transmissible Infections. COVID-19 = coronavirus disease 2019. FISABIO = Fundación para el Fomento de la Investigación Sanitaria y Biomédica. IQR = interquartile range. SARI = severe acute respiratory infection. SARS-CoV-2 = severe acute respiratory syndrome coronavirus 2.

Population demographics, clinical characteristics, and vaccination status of test-negative controls and test-positive cases.

a Totals sum to 100% across countries or across Study Contributors; for countries in which there was more than one Study Contributor, indented rows illustrate the numbers of participants from each Study Contributor within that country. The number of test-negative control participants from Austria, Klinik Favoriten, was limited due to logistical problems with accessing patient records.

b Participants with >1 chronic condition are included in each relevant category, and so the percentages for all categories sum to >100 within each column.

c As there is no linkage to national testing data, information about prior SARS-CoV-2 infection is reliant on hospital records and patient recall.

d Although disease onset occurred prior to the study period in some participants, these individuals were hospitalised within the study period.

e Cases for which samples were not genetically sequenced are classified as ‘missing’. Cases with sequenced samples but uninterpretable or unknown results are classified as ‘not interpretable/unknown’.

f None of the participants completed their two-dose primary series before March 2021 or after September 2021.

COVID-19 vaccine primary-series coverage increased over the analysis period; overall coverage rates for all COVID-19 vaccines in adults ranged from 25.2% (Austria) to 28.2% (Belgium) at the start of the study period (week 22, 2021; including June 1st) and from 83.7% (Austria) to 90.1% (Belgium) at the end of this analysis period (week 35, 2022; including September 5th).^19^ AZD1222 primary-series coverage ranged from 1.2% (Spain) to 3.6% (Austria) at the start of the study period and from 10.5% (Austria) to 15.3% (Belgium) at the end of this analysis period.^19^ Of the 120 (15.8%) participants who were fully vaccinated with two doses of AZD1222, the majority (90.0%) had received their second dose in June or July 2021 (Table 1); median time since last dose was 143 days (interquartile range [IQR], 106–163), which equates to 4.7 months (IQR, 3.5–5.4).

**Fig. 1:**
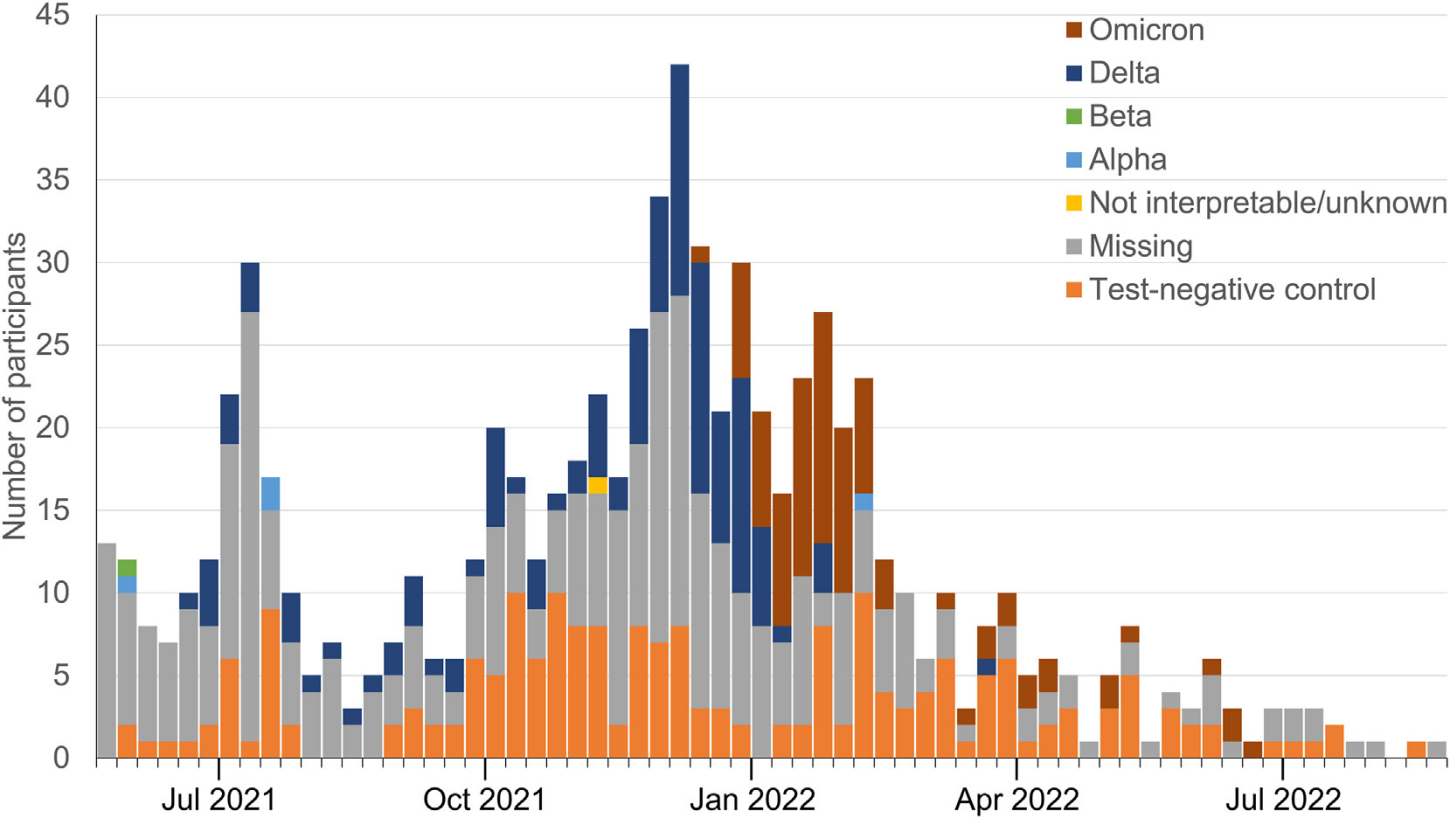
**Number of test-positive cases (by SARS-CoV-2 variant) and test-negative controls over the study period that were included in the AZD1222 VE analysis**. Cases for which samples were not genetically sequenced are classified as ‘missing’. Cases with sequenced samples but uninterpretable or unknown results are classified as ‘not interpretable/unknown’. Sequencing of positive test samples was not possible for retrospectively recruited patients. Data were also missing due to delays in sequencing for more recent cases. VE = vaccine effectiveness.

The dominant circulating SARS-CoV-2 variant of concern also changed over time during this analysis period, which began at the end of an Alpha-dominant wave and included a Delta-dominant wave and three separate Omicron subvariant-dominant waves in all four countries (https://covariants.org/per-country). For time-stratified analysis of VE by dominant variant, the analysis period was divided into five calendar time-windows coinciding with changes in the dominance of SARS-CoV-2 genetic variants as reported by the European Centre for Disease Prevention and Control (https://www.ecdc.europa.eu/en/covid-19/situation-updates/variants-dashboard) and covariants.org: July 1st to 31st, 2021 (Alpha, Delta variants dominant); August 1st to November 30th, 2021 (Delta); December 1st to 31st, 2021 (Delta, Omicron BA.1); January 1st to March 31st, 2022 (Omicron BA.1/BA.2); and April 1st to September 5th, 2022 (Omicron BA.2/BA.4/BA.5/BA.2.12.1/BA.2.75). The numbers of test-positive cases (by SARS-CoV-2 genetic variant) and test-negative controls over the study period by week of symptom onset are shown in Fig. 1. Participant enrolment peaked during November 2021 through January 2022, when 332 (43.6%) of the 761 hospitalisations due to SARIs occurred, including 271 (48.3%) of the 561 test-positive cases.

Participant characteristics according to timing of hospitalisation within the above five calendar time-windows are summarised in Supplementary Table S3. There was an increase in the median age of study participants from 55.0 years (IQR, 37.8–65.0) in those hospitalised in June–July 2021 (Alpha/Delta wave) to 70 years (IQR, 58.0–80.0) in those hospitalised between April 1st and September 5th, 2022 (mixed Omicron variants), primarily associated with the increasing median age of test-positive cases (from 50.0 to 72.0 years); the median age of test-negative controls was similar across time-windows (59.0–66.0 years). The percentage of participants hospitalised with SARI who had ≥1 chronic condition increased from 53.6% in June–July 2021 to 69.6% between April 1st and September 5th, 2022 (from 46.2% to 70.0% in test-positive cases); the overall proportion of participants with immunodeficiency was low and generally similar across the analysis period (3.7–12.2%; 2.9–8.9% in test-positive cases).

### Vaccine effectiveness estimates

**Fig. 2:**
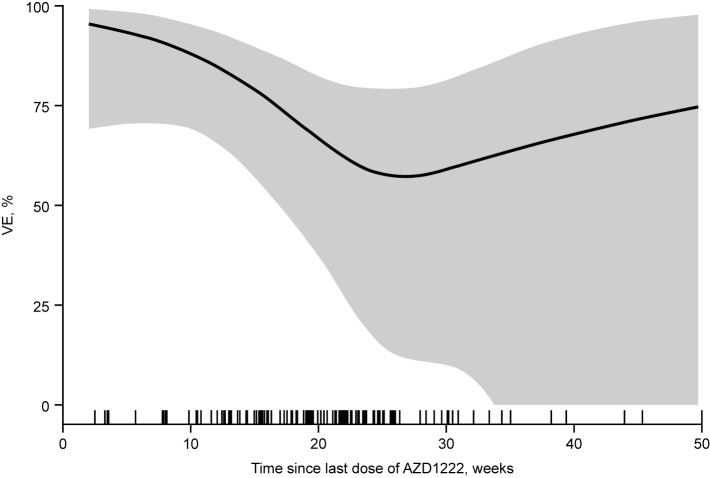
**Spline-based VE of two-dose primary-series AZD1222 vaccination against hospitalisation due to COVID-19 in SARI patients, by time since last dose of AZD1222**. The grey shaded area corresponds to the truncated 95% CI, and the tick-marks on the x-axis indicate the symptom onset dates (in relation to the date of the last vaccine dose) of the SARI patients included in the analysis. The rugs at the bottom of the plot have a small amount of noise (up to ±3 weeks) added to the actual values. CI = confidence interval. SARI = severe acute respiratory infection. VE = vaccine effectiveness.

In all individuals included during the 15-month analysis period from June 2021 to September 2022, the overall confounder-adjusted VE estimate against SARI hospitalisation due to COVID-19 was 72.8% (95% CI, 53.4 to 84.1; Table 2). Estimated VE showed some variation by time since last dose of AZD1222, with VE of 93.8% (48.6–99.3) at 2–8 weeks, 79.4% (41.9–92.7) at >8–16 weeks, and 67.2% (30.8–84.4) at >16–24 weeks (Table 2). The VE estimated in discrete time intervals (Table 2) has limited interpretation in this interim analysis as data for time intervals of >20 weeks have increasingly wide 95% CIs associated with decreasing numbers of events. The spline-based curve (Fig. 2) similarly demonstrates levels of protection conferred by primary-series AZD1222 by time since last dose, with protection at 30 weeks (VE of ≥50%), albeit with wide 95% CIs. With 90.0% of vaccinated participants having completed their AZD1222 primary series in June and July 2021, the cluster of hospitalisations occurring at ≥15–20 weeks after last dose (Fig. 2) are estimated to be due primarily to the Omicron variant among the test-positive cases. Confounder-adjusted VE estimates are supported by similar findings from symptom onset date-adjusted VE estimates (Supplementary Table S4). VE estimates by SARS-CoV-2 genetic variant could not be evaluated for the Alpha and Beta variants, and 95% CIs were generally very wide for VE estimates against the Delta and Omicron variants due to limited numbers of events with sequencing data available. VE estimates for cases in which the SARS-Cov-2 variant was uninterpretable, unknown, or not captured were broadly similar to the overall VE estimates (Table 3). Time-stratified analysis of VE by calendar time demonstrated a high initial VE estimate (91.0%) with waning over time, associated with an increasing mean time since last dose of AZD1222 (Supplementary Table S5). However, as with other stratified analyses at this interim analysis of COVIDRIVE, 95% CIs were very wide due to limited numbers of events.

**Table 2:** Confounder-adjusted VE estimates and cumulative confounder-adjusted VE estimates (adjusted for symptom onset, sex, age and chronic conditions) by time since last dose in SARI patients who had received two doses of AZD1222 and were hospitalised between June 1st, 2021, and September 5th, 2022.

Time since last dose of AZD1222	Exposure	Test-negative controls, n	Test-positive cases, n	VE, % (95% CI)
**Time period**				
2 to ≤8 weeks	Vaccinated	4	2	93.8 (48.6–99.3)
	Unvaccinated	140	501	
>8 to ≤16 weeks	Vaccinated	20	8	79.4 (41.9–92.7)
	Unvaccinated	140	501	
>16 to ≤24 weeks	Vaccinated	25	38	67.2 (30.8–84.4)
	Unvaccinated	140	501	
>24 to ≤32 weeks	Vaccinated	5	10	56.6 (−52.6 to 87.7)
	Unvaccinated	140	501	
>32 weeks	Vaccinated	6	2	76.1 (−39.7 to 95.9)
	Unvaccinated	140	501	
**Cumulative time**				
≤8 weeks	Vaccinated	4	2	93.8 (48.6–99.3)
	Unvaccinated	140	501	
≤16 weeks	Vaccinated	24	10	84.0 (59.1–93.8)
	Unvaccinated	140	501	
≤24 weeks	Vaccinated	49	48	74.5 (53.2–86.1)
	Unvaccinated	140	501	
≤32 weeks	Vaccinated	54	58	71.3 (49.6–83.6)
	Unvaccinated	140	501	
**All (full analysis period)**^a^	Vaccinated	60	60	72.8 (53.4–84.1)
	Unvaccinated	140	501	

CI = confidence interval. VE = vaccine effectiveness. SARI = severe acute respiratory infection.

a Estimated VE in all individuals included during the 15-month analysis period from June 2021 to September 2022, regardless of time since vaccination.

**Table 3:** Cumulative confounder-adjusted VE estimates (adjusted for symptom onset, sex, age and chronic conditions) by time since last dose and SARS-CoV-2 variant in SARI patients who had received two doses of AZD1222 and were hospitalised between June 1st, 2021, and September 5th, 2022.

Time since last dose of AZD1222	VE by SARS CoV-2 variant, % (95% CI)				
	Alpha	Beta	Delta	Omicron	Missing
**Time period**					
2 to ≤8 weeks	NE	NE	92.9 (−15.8 to 99.6)	NE	97.4 (62.0–99.8)
>8 to ≤16 weeks	NE	NE	78.8 (−1.2 to 95.6)	NE	84.3 (46.5–95.4)
>16 to ≤24 weeks	NE	NE	58.4 (−26.8 to 86.4)	74.8 (−584.7 to 99.1)	66.6 (24.0–85.4)
>24 to ≤32 weeks	NE	NE	87.9 (−73.3 to 99.2)	−26.9 (−622.3 to 77.7)	86.7 (6.7–98.1)
>32 weeks	NE	NE	NE	46.9 (−693.3 to 96.5)	76.8 (−132.1 to 97.7)
**Cumulative time**					
≤8 weeks	NE	NE	92.9 (−15.8 to 99.6)	NE	97.4 (62.0–99.8)
≤16 weeks	NE	NE	85.0 (41.3–96.2)	NE	89.1 (65.6–96.6)
≤24 weeks	NE	NE	71.0 (30.7–87.9)	74.8 (−584.9 to 99.1)	76.6 (53.3–88.3)
≤32 weeks	NE	NE	71.8 (34.0–87.9)	−9.5 (−462.6 to 78.7)	77.5 (55.9–88.5)
**All**	NE	NE	72.0 (34.7–88.0)	11.0 (−250.3 to 77.4)	78.1 (58.2–88.6)

CI = confidence interval. VE = vaccine effectiveness. NE = not estimable. SARI = severe acute respiratory infection. SARS-CoV-2 = severe acute respiratory syndrome coronavirus 2.

### Literature review and meta-regression analysis of AZD1222 VE against hospitalisation

**Fig. 3:**
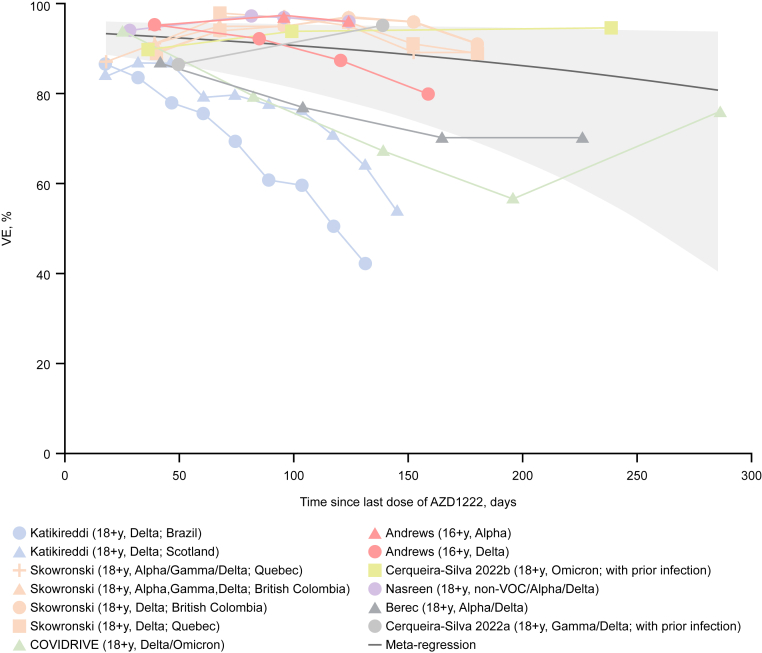
**VE of two-dose primary-series AZD1222 vaccination against hospitalisation due to COVID-19, by time since last dose of AZD1222 – meta-regression analysis**. VE shown from studies in the publicly available literature (as cited in Table 4) conducted in general populations, by SARS-CoV-2 variant, as well as from COVIDRIVE. Shaded area around meta-regression curve shows 95% confidence interval. COVID-19 = coronavirus disease 2019. VE = vaccine effectiveness.

The literature review identified seven publications reporting data on durability of VE of AZD1222 in general populations comprising adults of all ages (Table 4). A series of estimates from these publications of VE over time against different SARS-CoV-2 variants were incorporated in the meta-regression analysis along with the data from this interim analysis of COVIDRIVE to determine VE against severe disease over time in a population comprising all adults (Fig. 3). Results of the meta-regression were consistent with those from the present interim analysis of COVIDRIVE, with a high level of protection against hospitalisation due to COVID-19 that showed sustained protection through 43 weeks, albeit with a degree of waning observed and with increasingly wide 95% CIs.

**Table 4:** Observational studies included in meta-regression on durability of protection against hospitalisation following two-dose primary-series AZD1222 vaccination.

Publication	Study type	Country	Population	Study period	SARS-CoV-2 variants	VE timing relative to 2nd dose of AZD1222, d	Severe COVID-19/hospitalisation outcome(s)
Andrews, 2022^9^	TNCC (National register: NIMS, ECDS, NHS Digital)	UK	Aged ≥16 y with no prior COVID-19 diagnosis, with stratification by ≥16, 40–64, ≥65 y	Dec 8th, 2020, to Oct 1st, 2021	Delta (sequenced), Alpha (sequenced)	Range 14–≥140; max ∼180^a^	Hospital admission within 14 d of positive COVID-19 test, with COVID-19 or respiratory SNOMED CT codes
Berec, 2022^20^	Cohort (National database ISID)	Czechia	Aged ≥18 y, regardless of prior COVID-19 diagnosis	Dec 26th, 2020, to Nov 20th, 2021	Alpha, Delta (predominant variant)	Range 14–257	Hospital admission with positive PCR test within 2 weeks before or at any time during hospitalisation
Cerquira-Silva, 2022^b^^,^^21^	TNCC (National databases SI–PNI, e-SUS-Notifica and SIVEP-Gripe)	Brazil	Aged ≥18 y with reported COVID-19 symptoms and tests Median (IQR) age, y: Cases: 37 (29–48) Controls: 36 (27–48) Overall: 37 (28–48)	Jan 1st to Mar 22nd, 2022	Omicron (predominant variant)	Range 15–≥140; max 339^b^	Positive test from 14 d before to 3 d after hospital admission or death occurring within 28 d after positive test
Cerqueira-Silva, 2022^a^^,^^22^	TNCC (National databases SI–PNI, e-SUS-Notifica and SIVEP-Gripe)	Brazil	Aged ≥18 y with prior SARS-CoV-2 infection Median (IQR) age, y: Cases: 37 (29–46) Controls: 36 (29–44)	Feb 24th, 2020, to Nov 11th, 2021	Non-VoC, Gamma, Delta (predominant variant)	Range 0–≥91; max ∼190^c^	Hospital admission with positive SARS-CoV-2 RT-PCR test within 28 days of sample collection date
COVIDRIVE Study	TNCC (Hospital databases)	Austria, Belgium, Italy, Spain	Hospitalised SARI patients aged ≥18 y with specimen collected for RT-PCR test	Jun 1st, 2021, to Sep 5th, 2022	Delta, Omicron (predominant variant, also by sequencing)	Range 0–348	Hospitalised with SARI and ≥1 positive test for SARS-CoV-2 on RT-PCR or similar molecular assay with samples collected between 14 d prior to and the day of hospital admission
Katikireddi, 2022^10^	Cohort (EAVE II study in Scotland, and three national databases, SI–PNI, e-SUS-Notifica and SIVEP-Gripe, in Brazil)	Scotland, Brazil	Aged ≥18 y with no prior COVID-19 diagnosis Median (IQR) age, y: Scotland: 57 (48–68) Brazil: 51 (40–60)	May 19th to Oct 25th, 2021 (Scotland) Jan 18th to Oct 25th, 2021 (Brazil)	Delta (predominant variant)	Range 0–153	Hospital admission or death as defined in databases with COVID-19 code (ICD, 10th revision: U07.1, U07.2)
Nasreen, 2022^23^	TNCC (Provincial databases)	Canada	Aged ≥18 y regardless of prior COVID-19 diagnosis Median (IQR) age, y: British Columbia: 61 (52–72) Manitoba: 38 (31–46) Ontario: 48 (35–62) Quebec: 63 (56–78)	Dec 14th, 2020, to Sep 30th, 2021	Non-VoC, Alpha, Beta, Gamma, Delta (predominant variant)	Range 7–111	Positive test from 14 d before to 3 d after hospital admission; or death occurring within 30 d after positive test or within 7 d post-mortem
Skowronski, 2022^12^	TNCC (Provincial databases)	Canada (British Columbia, Quebec)	Aged ≥18 y with specimen collection for NAAT test Median (IQR) age, y: British Columbia: Cases: 39 (29–54) Controls: 42 (31–58) Quebec: Cases: 40 (29–52) Controls: 44 (32–61)	May 30th to Nov 27th, 2021	Alpha, Gamma, Delta (predominant variant), Delta (sequenced)	Range 0–195	Hospital admission on or within 30 d after specimen collection

COVID-19 = coronavirus disease 2019. d = days. EAVE II = Early Pandemic Evaluation and Enhanced Surveillance of COVID-19 study. ECDS = Emergency Care Data Set. e-SUS-Notifica = Brazilian Acute Respiratory Infection Suspected Cases dataset. ICD = International Classification of Diseases. IQR = interquartile range. ISID = Information System of Infectious Diseases. NAAT = nucleic acid amplification test. NHS = National Health Service. NIMS = National Immunisation Management System. (RT)-PCR = (reverse transcription) polymerase chain reaction. RTI = respiratory tract infection. SARI = severe acute respiratory infection. SARS-CoV-2 = severe acute respiratory syndrome coronavirus 2. SI–PNI = Brazilian COVID-19 Vaccination Campaign dataset. SIVEP-Gripe = Brazilian Severe Acute Respiratory Infection/Illness dataset. SNOMED CT = Systematised Nomenclature of Medicine–Clinical Terms. TNCC = test-negative case–control design. VE = vaccine effectiveness. VoC = variant of concern. wks = weeks. y = years.

a Reported for entire study population.

b Maximum time calculated based on Brazilian data on AZD1222 vaccine roll-out and study end date.

c Determined from data shown in supplementary Figure A3 of the cited publication.

## Discussion

These results from the prespecified second interim analysis of the COVIDRIVE AZD1222-specific study demonstrate the VE and duration of protection conferred by primary-series AZD1222 vaccination against hospitalisation due to COVID-19. An overall confounder-adjusted VE estimate of 72.8% (95% CI, 53.4 to 84.1), with a median time since vaccination of 20 weeks, was determined for the 15-month analysis period (June 1st, 2021–September 5th, 2022) during which Delta followed by Omicron were the predominant variants. For the time-period up to 24 weeks post-primary series, this estimate was 74.5%, with data suggesting ongoing effectiveness after longer follow-up but with limited certainty and wide 95% CIs for the point estimates of VE estimated in discrete time intervals. Protection conferred over time since primary series was seen on spline curve analysis, with protection potentially beyond 30 weeks but with 95% CIs that cross 0 after 35 weeks. The findings of this interim analysis will need confirming at the final analysis with a greater population size. Nevertheless, our data suggest that individuals who received a complete primary-series vaccination with AZD1222 but opted not to or were unable to receive COVID-19 vaccine booster dosing potentially retained a level of protection against severe COVID-19 outcomes due to their primary-series vaccination, even during a period when Omicron was the dominant variant.

Reflecting evidence from the pivotal phase III AZD1222 study^7^ and from other real-world effectiveness studies,^9^^,^^12^ these COVIDRIVE data also indicate some degree of waning at approximately 6 months; however, it is important to note that for a majority of participants in COVIDRIVE, 6 months post-second dose coincided with the rapid increase in incidence of infections associated with the first Omicron-driven wave. Our literature review and meta-regression analysis on the durability of protection against COVID-19-related hospitalisation conferred by primary-series AZD1222 in the general adult population, which incorporated effectiveness studies that were mostly conducted during the period when Delta was the dominant variant,^10^^,^^12^^,^^20^, ^21^, ^22^, ^23^ suggested that AZD1222 VE against hospitalisation remains high (>80%) for up to 300 days (approximately 43 weeks) after the completion of the primary series, albeit with increasingly wide CIs. There is evidence to indicate that VE against symptomatic disease due to Omicron and its subvariants is lower than for Delta^24^; however, data from booster studies suggest that vaccination maintains high levels of protection against severe outcomes due to both Delta and Omicron.^25^^,^^26^

Our findings on the effectiveness of AZD1222 against hospitalisation are supported by clinical trial data demonstrating the durability of humoral and cellular immunity conferred by primary-series AZD1222 vaccination.^7^^,^^27^ Sustained T-cell responses across SARS-CoV-2 variants have been shown to be important in conferring durable protection against severe disease.^28^

A strength of the COVIDRIVE study is that RT-PCR of nasopharyngeal swabs was used in testing for SARS-CoV-2, which has high specificity and sensitivity and thus minimises the potential for misclassification of test-positive cases and test-negative controls. Another strength is that the findings are based on the real-world use of AZD1222 in multiple EU countries. However, this also gives rise to some limitations, including high levels of VE heterogeneity observed between Study Contributor level (data not shown), as well as the differing and changing vaccination recommendations for use of AZD1222 in the different participating countries. For example, the median age of study participants recruited in Belgium was only 49.0 years, compared with 57.0–63.0 years in the other countries (Supplementary Table S2), illustrating the differences in prioritisation and roll-out by country. Additionally, with the aim of reducing participant and exposure heterogeneity, the analysis was restricted to those who had received homologous two-dose primary-series vaccination with AZD1222 and to participants with symptom onset within 14 days prior to hospitalisation, which resulted in a limited sample size and thus very wide CIs for some VE estimates. A further limitation is the potential presence of residual confounding, which is especially important in COVID-19 VE studies as confounding factors remain unknown or are difficult to take into account. For example, COVIDRIVE was not designed to capture prior SARS-CoV-2 infection systematically, as this would have required (repeated) serological testing, which would have implied a very resource-intensive study with medical interventions outside of routine care, and so the potential effect of prior natural infection on VE (conferring some degree of protection in unvaccinated individuals and greater protection through hybrid immunity in vaccinated individuals) could not be assessed as data availability was limited. Further, without robust information on prior infection, individuals could not be excluded from the analysis on the basis of this potential confounding factor; nevertheless, our estimates reflect real-world VE in the context of current immunity levels in the population. There was also a high level of missing data on SARS-CoV-2 variant sequencing, and no stratification by age. Confounding due to unmeasured factors, such as education and socioeconomic status, which influence COVID-19 vaccination status, is also likely; similarly, confounding could also have occurred due to imbalances in other factors that may influence COVID-19 vaccination status, such as influenza and pneumococcal vaccination status, but that we did not adjust for in these analyses. Additionally, it is possible that body mass index, which we could not sufficiently measure, affected our estimates. Further, our analysis has underrepresentation of long-term care facility residents, and so the VE of AZD1222 in real-world use may be lower than found in this analysis. Finally, our analysis excluded participants who had received partial or heterologous primary-series vaccination in order to determine the VE specifically associated with the recommended two-dose primary-series schedule of AZD1222, and no data on VE following booster doses have been included as the aim was to focus on the durability of protection among those who opted not to or were unable to receive a booster. However, the application of these exclusion criteria was important for stringently defining the scope of our literature review and meta-regression and ensuring a consistent population with consistent AZD1222 exposure. A limitation of our focused approach was that only studies in the general population were included, whereas VE in high-risk populations such as immunocompromised individuals may be impacted by lower overall effectiveness and more rapid waning.^29^^,^^30^ Our meta-regression may also overestimate VE against currently circulating variants because the majority of studies of AZD1222 VE durability available in the public domain describe VE durability against the Delta variant, rather than Omicron or its more recent subvariants, which may more readily evade protection.

In conclusion, these interim analysis findings from COVIDRIVE provide further support for the important ongoing role played by AZD1222 in the global pandemic response, demonstrating substantial VE against hospitalisation and the level of protection conferred over time. Consequently, and because it is easy to store and distribute, AZD1222 represents a valuable COVID-19 vaccine option for use in some countries and regions, particularly for resource-constrained low- and middle-income countries with lower vaccination coverage and/or challenges to implementing rapid mass vaccination programmes. Our findings also provide evidence for a level of long-term protection against severe COVID-19 outcomes following primary-series AZD1222 vaccination. The COVIDRIVE study is ongoing, with additional prospective and retrospective data collection and sequencing of samples, and the final analysis of AZD1222-specific data will thus provide confirmatory evidence in a larger population.

## Contributors

This AZD1222-specific study was designed by members of the COVIDRIVE consortium in collaboration with the AZD1222 MAH, AstraZeneca. LD, GR and AC are involved in the coordination of the COVIDRIVE study. GM, AM-I, GI, SO-R, SB and CM are principal investigators at the Study Contributors (study sites) and were responsible for data collection and coordination at their respective sites. Investigators gathered the data in collaboration with P95 and AstraZeneca. The data were analysed/interpreted by WM, LdM, CW-T, MO, ST and KB and statistical analysis was performed by AD. The accuracy of the data was verified by WM, LdM, and KB. The literature review was conducted by ALS and TYCN and reviewed by WM; the meta-regression was conducted by CMG. The manuscript was written under the direction of all authors by medical writers funded by AstraZeneca. All authors reviewed and provided feedback on the manuscript drafts and approved the manuscript for submission.

## Data sharing statement

Data underlying the findings described in this manuscript may be obtained in accordance with AstraZeneca’s data sharing policy described at https://astrazenecagrouptrials.pharmacm.com/ST/Submission/Disclosure. Data for studies directly listed on Vivli can be requested through Vivli at www.vivli.org (with hyperlink= https://www.vivli.org). Data for studies not listed on Vivli could be requested through Vivli at https://vivli.org/members/enquiries-about-studies-not-listed-on-the-vivli-platform/. AstraZeneca Vivli member page is also available outlining further details: https://vivli.org/ourmember/astrazeneca/.

## Declaration of interests

**WM, CMG, MO, and ST** declare employment by AstraZeneca and ownership of AstraZeneca stocks/shares.

**LdM, AD, CW-T, WH-M, LD, GR, ALS, TYCN, and KB** declare employment by P95. **ALS** and **TYCN** were contracted to AstraZeneca at the time of this work. P95 received consulting fees from several COVID-19 vaccine manufacturers, including AstraZeneca, for the COVIDRIVE study.

**AC** declares funding from COVIDRIVE industry partners (AstraZeneca, Janssen, Moderna, Novavax, CureVac, Sanofi, Valneva, GlaxoSmithKline, Bavarian Nordic) for the COVIDRIVE consortium, of which FISABIO is the coordinator, and honoraria for lectures and educational events from GlaxoSmithKline and for presentations from MSD and HIPRA.

**GM** declares no conflicts of interest related to this analysis, and honoraria from GlaxoSmithKline associated with herpes virus vaccine.

**AM-I** declares funding from COVIDRIVE industry partners (AstraZeneca, Janssen, Moderna, Novavax, CureVac, Sanofi, Valneva, GlaxoSmithKline, Bavarian Nordic) for the COVIDRIVE consortium, of which FISABIO is the coordinator, and honoraria for educational events from MSD and for presentations from Sanofi Pasteur.

**GI** declares no conflicts of interest.

**SO-R** declares payment or honoraria for lectures, presentations, speakers bureaus, manuscript writing or educational events from EXCEMED and Sanofi.

**SB** declares speaker honorarium from GlaxoSmithKline.

**CM** declares advisory board participation for AstraZeneca in 2021.

## Appendix A Supplementary data

Supplementary Figure S1 and Tables S1–S5, and COVIDRIVE master protocol
