# Supplementary material for: Protection against COVID-19 hospitalisation conferred by primary-series vaccination with AZD1222 in non-boosted individuals: first vaccine effectiveness results of the European COVIDRIVE study and meta-regression analysis
Source: Lancet Reg Health Eur. 2023 Jul 1;31:100675. doi: 10.1016/j.lanepe.2023.100675 (PMC10398604; doi:10.1016/j.lanepe.2023.100675)
Supplement: Supplementary Figure S1 and Tables S1–S5, and COVIDRIVE master protocol [file mmc1.pdf]

Supplementary Appendix

Table of contents

**Content**	**Page**
**Supplemental Tables**	
Table S1: Recruitment periods for the Study Contributors (databases) from which data for the analysis were received	2
Table S2: Demographic characteristics of the study population, stratified by country	3
Table S3: Demographic characteristics of the study population, stratified by SARS-CoV-2 variant periods	6
Table S4: Cumulative symptom onset date-adjusted VE estimates by time since last dose and by SARS-CoV-2 genetic variant in SARI patients who had received two doses of AZD1222	9
Table S5: Confounder-adjusted VE estimates by calendar time in SARI patients who had received two doses of AZD1222 and were hospitalised between June 1, 2021 and September 5, 2022	10
**Supplemental Figures**	
Figure S1: Attrition diagram of participants in the COVIDRIVE study and the SARI patients in this study of AZD1222.	11

COVIDRIVE master protocol

The COVIDRIVE master protocol is registered with the European Network of Centres for Pharmacoepidemiology and Pharmacovigilance (ENCePP, EUPAS42328) and is available to download from: https://www.encepp.eu/encepp/viewResource.htm?id=42329 – the protocol is also available to download from the COVIDRIVE study website at: https://covidrive.eu/study-documents/, including current and previous versions.

Table S1: Recruitment periods for the Study Contributors (databases) from which data for the analysis were received.

Country	Study Contributor	Number of individual hospitals, n	Start of prospective recruitment[a]	Start of retrospective recruitment[b]
Austria	Klinik Favoriten, Vienna	1	April 4th, 2022	June 1st, 2021[c]
Belgium	Le Centre Hospitalier Universitaire St Pierre, Brussels	1	February 3rd, 2022	June 1st, 2021[c]
Belgium	Universitair Ziekenhuis Antwerpen (UZA), Antwerp	1	December 28th, 2021	June 1st, 2021
Italy	Inter-university Research Centre on Influenza and Other Transmissible Infections (CIRI-IT), Genoa	4	November 16th, 2021	NA[d]
Spain	Fundación para el Fomento de la Investigación Sanitaria y Biomédica (FISABIO) de la Comunitat Valenciana, Salud Pública, Valencia	4	September 15th, 2021	NA[d]
Spain	Hospital Germans Trias i Pujol, Badalona, Barcelona	1	October 20th, 2021	July 1st, 2021[c]
Spain	Hospital Universitari Vall d'Hebron, Barcelona	1[e]	February 14th, 2022	October 30th, 2021

[a]Start of prospective patient recruitment is the date the site opened for recruitment. [b]Date of hospital admission of the first admitted patient among the retrospective data. [c]Study Contributor yet to provide all retrospective data. [d]Study Contributor is not contracted to collect retrospective data and thus only provided prospective data. [e]Hospital Universitari Vall d'Hebron only provided data from one hospital for this interim analysis but a second hospital is joining COVIDRIVE at this Study Contributor.
NA=not applicable.

***Table S2*: Demographic characteristics of the study population, stratified by country**

Characteristic	Austria (Jun 2021–Sep 2022)			Belgium (Jun 2021–Sep 2022)			Italy (Nov 2021–Sep 2022)			Spain (Jul 2021–Sep 2022)		
	**Total (N=79)**	**TNC (n=2)**	**TPC (n=77)**	**Total (N=153)**	**TNC (n=24)**	**TPC (n=129)**	**Total (N=131)**	**TNC (n=36)**	**TPC (n=95)**	**Total (N=398)**	**TNC (n=138)**	**TPC (n=260)**
**Age at hospitalisation, years**	57·0 (44·0–68·5)	79·5 (77·8–81·2)	56·0 (44·0–68·0)	49·0 (37·0–63·0)	58·5 (34·8–73·8)	49·0 (37·0–60·0)	63·0 (51·5–72·5)	59·0 (45·8–68·5)	65·0 (54·0–74·0)	62·0 (44·2–69·0)	64·0 (54·0–69·0)	61·0 (43·0–69·0)
18–49	29 (36·7)	0	29 (37·7)	78 (51·0)	10 (41·7)	68 (52·7)	26 (19·8)	13 (36·1)	13 (13·7)	115 (28·9)	30 (21·7)	85 (32·7)
50–64	25 (31·6)	0	25 (32·5)	38 (24·8)	4 (16·7)	34 (26·4)	43 (32·8)	10 (27·8)	33 (34·7)	119 (29·9)	40 (29·0)	79 (30·4)
≥65	25 (31·6)	2 (100)	23 (29·9)	37 (24·2)	10 (41·7)	27 (20·9)	62 (47·3)	13 (36·1)	49 (51·6)	164 (41·2)	68 (49·3)	96 (36·9)
**Sex**												
Female	40 (50·6)	1 (50·0)	39 (50·6)	63 (41·2)	9 (37·5)	54 (41·9)	55 (42·0)	14 (38·9)	41 (43·2)	165 (41·5)	54 (39·1)	111 (42·7)
Male	39 (49·4)	1 (50·0)	38 (49·4)	90 (58·8)	15 (62·5))	75 (58·1)	76 (58·0)	22 (61·1)	54 (56·8)	233 (58·5)	84 (60·9)	149 (57·3)
**Body mass index (BMI) category**												
Underweight (BMI <18·5)	1 (1·3)	0	1 (1·3)	2 (1·3)	0	2 (1·6)	2 (1·5)	1 (2·8)	1 (1·1)	1 (0·3)	0	1 (0·4)
Normal/healthy weight (BMI ≥18·5 to <25·0)	12 (15·2)	0	12 (15·6)	9 (5·9)	3 (12·5)	6 (4·7)	52 (39·7)	18 (50·0)	34 (35·8)	51 (12·8)	23 (16·7)	28 (10·8)
Overweight (BMI ≥25·0 to <30·0)	24 (30·4)	1 (50·0)	23 (29·9)	15 (9·8)	1 (4·2)	14 (10·9)	39 (29·8)	12 (33·3)	27 (28·4)	54 (13·6)	19 (13·8)	35 (13·5)
Obese (BMI ≥30·0)	37 (46·8)	1 (50·0)	36 (46·8)	9 (5·9)	1 (4·2)	8 (6·2)	24 (18·3)	5 (13·9)	19 (20·0)	58 (14·6)	17 (12·3)	41 (15·8)
No information	0	0	0	0	0	0	0	0	0	7 (1·8)	1 (0·7)	6 (2·3)
Missing	5 (6·3)	0	5 (6·5)	118 (77·1)	19 (79·2)	99 (76·7)	14 (10·7)	0	14 (14·7)	227 (57·0)	78 (56·5)	149 (57·3)
**Number of chronic conditions**												
0	27 (34·2)	0	27 (35·1)	82 (53·6)	7 (29·2)	75 (58·1)	61 (46·6)	17 (47·2)	44 (46·3)	149 (37·4)	30 (21·7)	119 (45·8)
1	14 (17·7)	0	14 (18·2)	23 (15·0)	6 (25·0)	17 (13·2)	30 (22·9)	9 (25·0)	21 (22·1)	89 (22·4)	29 (21·0)	60 (23·1)
2	14 (17·7)	0	14 (18·2)	23 (15·0)	4 (16·7)	19 (14·7)	23 (17·6)	7 (19·4)	16 (16·8)	79 (19·8)	35 (25·4)	44 (16·9)
≥3	24 (30·4)	2 (100)	22 (28·6)	25 (16·3)	7 (29·2)	18 (14·0)	17 (13·0)	3 (8·3)	14 (14·7)	81 (20·4)	44 (31·9)	37 (14·2)
**Participants with chronic condition[a]**												
Asthma	6 (7·6)	0	6 (7·8)	8 (5·2)	4 (16·7)	4 (3·1)	5 (3·8)	1 (2·8)	4 (4·2)	29 (7·3)	17 (12·3)	12 (4·6%)
Lung disease	14 (17·7)	1 (50·0)	13 (16·9)	22 (14·4)	8 (33·3)	14 (10·9)	17 (13·0)	7 (19·4)	10 (10·5)	85 (21·4)	47 (34·1)	38 (14·6)
Cardiovascular disease	20 (25·3)	2 (100)	18 (23·4)	24 (15·7)	7 (29·2)	17 (13·2)	32 (24·4)	9 (25·0)	23 (24·2)	90 (22·6)	45 (32·6)	45 (17·3)
Hypertension	47 (59·5)	2 (100)	45 (58·4)	27 (17·6)	7 (29·1)	20 (15·5)	40 (30·5)	7 (19·4)	33 (34·7)	138 (34·7)	53 (38·4)	85 (32·7)
Chronic liver disease	4 (5·1)	0	4 (5·2)	8 (5·2)	1 (4·2)	7 (5·4)	4 (3·1)	1 (2·8)	3 (3·2)	16 (4·0)	7 (5·1)	9 (3·5)
Chronic kidney disease	9 (11·4)	0	9 (11·7)	17 (11·1)	5 (20·8)	12 (9·3)	6 (4·6)	2 (5·6)	4 (4·2)	36 (9·0)	19 (13·8)	17 (6·5)
Type 2 diabetes	23 (29·1)	0	23 (29·9)	26 (17·0)	4 (16·7)	22 (17·1)	16 (12·2)	1 (2·8)	15 (15·8)	68 (17·1)	24 (17·4)	44 (16·9)
Cancer	4 (5·1)	1 (50·0)	3 (3·9)	15 (9·8)	3 (12·5)	12 (9·3)	13 (9·9)	5 (13·9)	8 (8·4)	39 (9·8)	25 (18·1)	14 (5·4)
Immunodeficiency (or organ transplant)	5 (6·3)	1 (50·0)	4 (5·2)	18 (11·8)	2 (8·3)	16 (12·4)	2 (1·5)	1 (2·8)	1 (1·1)	28 (7·0)	20 (14·5)	8 (3·1)
**Pregnancy**	n=40	n=1	n=39	n=63	n=9	n=54	n=55	n=14	n=41	n=165	n=54	n=111
No	29 (72·5)	1 (100)	28 (71·8)	56 (88·9)	8 (88·9)	48 (88·9)	53 (96·4)	13 (92·9)	40 (97·6)	150 (90·9)	54 (100)	96 (86·5)

Characteristic	Austria (Jun 2021–Sep 2022) Total (N=79)	Austria TNC (n=2)	Austria TPC (n=77)	Belgium (Jun 2021–Sep 2022) Total (N=153)	Belgium TNC (n=24)	Belgium TPC (n=129)	Italy (Nov 2021–Sep 2022) Total (N=131)	Italy TNC (n=36)	Italy TPC (n=95)	Spain (Jul 2021–Sep 2022) Total (N=398)	Spain TNC (n=138)	Spain TPC (n=260)
Yes	2 (5·0)	0	2 (5·1)	7 (11·1)	1 (11·1)	6 (11·1)	1 (1·8)	0	1 (2·4)	12 (7·3)	0	12 (10·8)
No information	9 (22·5)	0	9 (23·1)	0	0	0	1 (1·8)	1 (7·1)	0	0	0	0
Missing	0	0	0	0	0	0	0	0	0	3 (1·8)	0	3 (2·7)
**Prior SARS-CoV-2 infection**[b]												
No prior infection	78 (98·7)	2 (100)	76 (98·7)	145 (94·8)	21 (87·5)	124 (96·1)	123 (93·9)	33 (91·7)	90 (94·7)	157 (39·4)	64 (46·4)	93 (35·8)
Yes – clinical diagnosis	0	0	0	1 (0·7)	0	1 (0·8)	2 (1·5)	1 (2·8)	1 (1·1)	0	0	0
Yes – laboratory-confirmed	1 (1·3)	0	1 (1·3)	2 (1·3))	1 (4·2)	1 (0·8)	4 (3·1)	2 (5·6)	2 (2·1)	12 (3·0)	10 (7·2)	2 (0·8)
No information	0	0	0	0	0	0	2 (1·5)	0	2 (2·1)	0	0	0
Missing	0	0	0	5 (3·3)	2 (8·3)	3 (2·3)	0	0	0	229 (57·5)	64 (46·4)	165 (63·5)
**Vaccinated for influenza within 12 months prior to SARI hospital admission**												
No	13 (16·5)	1 (50·0)	12 (15·6)	51 (33·3)	16 (66·7)	35 (27·1)	117 (89·3)	32 (88·9)	85 (89·5)	304 (76·4)	95 (68·8)	209 (80·4)
Yes	1 (1·3)	1 (50·0)	0	5 (3·3)	1 (4·2)	4 (3·1)	9 (6·9)	3 (8·3)	6 (6·3)	57 (14·3)	30 (21·7)	27 (10·4)
No information	65 (82·3)	0	65 (84·4)	97 (63·4)	7 (29·2)	90 (69·8)	5 (3·8)	1 (2·8)	4 (4·2)	18 (4·5)	11 (8·0)	7 (2·7)
Missing	0	0	0	0	0	0	0	0	0	19 (4·8)	2 (1·4)	17 (6·5)
**Received any pneumococcal vaccine**												
No	8 (10·1)	1 (50·0)	7 (9·1)	51 (33·3)	14 (58·3)	37 (28·7)	122 (93·1)	33 (91·7)	89 (93·7)	302 (75·9)	91 (65·9)	211 (81·2)
Yes	1 (1·3)	1 (50·0)	0	6 (3·9)	3 (12·5)	3 (2·3)	1 (0·8)	1 (2·8)	0	58 (14·6)	34 (24·6)	24 (9·2)
No information	70 (88·6)	0	70 (90·9)	96 (62·7)	7 (29·2)	89 (69·0)	8 (6·1)	2 (5·6)	6 (6·3)	20 (5·0)	11 (8·0)	9 (3·5)
Missing	0	0	0	0	0	0	0	0	0	18 (4·5)	2 (1·4)	16 (6·2)
**Smoking history**												
Never smoked	22 (27·8)	0	22 (28·6)	31 (20·3)	5 (20·8)	26 (20·2)	44 (33·6)	3 (8·3)	41 (43·2)	161 (10·5)	39 (28·3)	122 (46·9)
Ex-smoker	7 (8·9)	2 (100)	5 (6·5)	12 (7·8)	3 (12·5)	9 (7·0)	27 (20·6)	2 (5·6)	25 (26·3)	104 (26·1)	42 (30·4)	62 (23·8)
Occasional smoker	1 (1·3)	0	1 (1·3)	2 (1·3)	0	2 (1·6)	2 (1·5)	1 (2·8)	1 (1·1)	8 (2·0)	6 (4·3)	2 (0·8)
Daily smoker	8 (10·1)	0	8 (10·4)	12 (7·8)	7 (29·2)	5 (3·9)	15 (11·5)	5 (13·9)	10 (10·5)	53 (13·3)	36 (26·1)	17 (6·5)
No information	41 (51·9)	0	41 (53·2)	94 (61·4)	8 (33·3)	86 (66·7)	43 (32·8)	25 (69·4)	18 (18·9)	65 (16·3)	12 (8·7)	53 (20·4)
Missing	0	0	0	2 (1·3)	1 (4·2)	1 (0·8)	0	0	0	7 (1·8)	3 (2·2)	4 (1·5)
**Long-term care facility resident**												
No	77 (97·5)	2 (100)	75 (97·4)	148 (95·7)	23 (95·8)	125 (96·9)	130 (99·2)	36 (100)	94 (98·9)	382 (96·0)	129 (93·5)	253 (97·3)
Yes	1 (1·3)	0	1 (1·3)	1 (0·7)	0	1 (0·8)	0	0	0	2 (0·5)	1 (0·7)	1 (0·4)
No information	1 (1·3)	0	1 (1·3)	2 (1·3)	0	2 (1·6)	1 (0·8)	0	1 (1·1)	7 (1·8)	5 (3·6)	2 (0·8)
Missing	0	0	0	2 (1·3)	1 (4·2)	1 (0·8)	0	0	0	7 (1·8)	3 (2·2)	4 (1·5)
**Healthcare worker**												
No	71 (89·9)	2 (100)	69 (89·6)	137 (89·5)	22 (91·7)	115 (89·1)	130 (99·2)	36 (100)	94 (98·9)	245 (61·6)	88 (63·8)	157 (60·4)

Characteristic	Austria (Jun 2021–Sep 2022) Total (N=79)	TNC (n=2)	TPC (n=77)	Belgium (Jun 2021–Sep 2022) Total (N=153)	TNC (n=24)	TPC (n=129)	Italy (Nov 2021–Sep 2022) Total (N=131)	TNC (n=36)	TPC (n=95)	Spain (Jul 2021–Sep 2022) Total (N=398)	TNC (n=138)	TPC (n=260)
Yes	2 (2·5)	0	2 (2·6)	0	0	0	0	0	0	0	0	0
No information	6 (7·6)	0	6 (7·8)	13 (8·5)	1 (4·2)	12 (9·3)	1 (0·8)	0	1 (1·1)	147 (36·9)	47 (34·1)	100 (38·5)
Missing	0	0	0	3 (2·0)	1 (4·2)	2 (1·6)	0	0	0	6 (1·5)	3 (2·2)	3 (1·2)
**Period of disease onset**												
May–Jun 2021	37 (46·8)	0	37 (48·1)	10 (6·5)	3 (12·5)	7 (5·4)	0	0	0	8 (2·0)	2 (1·4)	6 (2·3)
Jul–Sep 2021	19 (24·1)	0	19 (24·7)	56 (36·6)	9 (37·5)	47 (36·4)	0	0	0	67 (16·8)	23 (16·7)	44 (16·9)
Oct–Dec 2021	0	0	0	71 (46·4)	10 (41·7)	61 (47·3)	34 (26·0)	1 (2·8)	33 (34·7)	202 (50·8)	72 (52·2)	130 (50·0)
Jan–Mar 2022	10 (12·7)	0	10 (13·0)	15 (9·8)	1 (4·2)	14 (10·9)	76 (58·0)	27 (75·0)	49 (51·6)	87 (21·9)	23 (16·7)	64 (24·6)
Apr–Jun 2022	9 (11·4)	2 (100)	7 (9·1)	0	0	0	17 (13·0)	8 (22·2)	9 (9·5)	29 (7·3)	14 (10·1)	15 (5·8)
Jul–Aug 2022	4 (5·1)	0	4 (5·2)	1 (0·7)	1 (4·2)	0	4 (3·1)	0	4 (4·2)	5 (1·3)	4 (2·9)	1 (0·4)
**Genetic variant**	n=77	n/a	n=77	n=129	n/a	n=129	n=95	n/a	n=95	n=260	n/a	n=260
Alpha	0	0	0	1 (0·8)	0	1 (0·8)	0	0	0	0	0	0
Beta	1 (1·3)	0	1 (1·3)	0	0	0	0	0	0	0	0	0
Delta	0	0	0	25 (19·4)	0	25 (19·4)	19 (20·0)	0	19 (20·0)	79 (30·4)	0	79 (30·4)
Omicron	0	0	0	6 (4·7)	0	6 (4·7)	26 (27·4)	0	26 (27·4)	54 (20·8)	0	54 (20·8)
Not interpretable/unknown	0	0	0	0	0	0	0	0	0	2 (0·8)	0	2 (0·8)
Missing	76 (98·7)	0	76 (98·7)	97 (75·2)	0	97 (75·2)	50 (52·6)	0	50 (52·6)	125 (48·1)	0	125 (48·1)
**Vaccinated against COVID-19**												
No	79 (100)	2 (100)	77 (100)	136 (88·9)	18 (75·0)	118 (91·5)	119	32 (88·9)	87 (91·6)	307 (77·1)	88 (63·8)	219 (84·2)
Fully vaccinated with two doses of AZD1222	0	0	0	17 (11·1)	6 (25·0)	11 (8·5)	12	4 (11·1)	8 (8·4)	91 (22·9)	50 (36·2)	41 (15·8)
**Month and year of second AZD1222 dose**	n=0	n=0	n=0	n=17	n=6	n=11	n=12	n=4	n=8	n=91	n=50	n=41
March 2021	0	0	0	1 (5·9)	0	1 (9·1)	0	0	0	0	0	0
April 2021	0	0	0	0	0	0	0	0	0	0	0	0
May 2021	0	0	0	2 (11·8)	1 (16·7)	1 (9·1)	3	2 (50·0)	1 (12·5)	0	0	0
June 2021	0	0	0	5 (29·4)	3 (50·0)	2 (18·2)	1	0	1 (12·5)	27 (29·7)	16 (32·0)	11 (26·8)
July 2021	0	0	0	8 (47·1)	1 (16·7)	7 (63·6)	6	1 (25·0)	5 (62·5)	61 (67·0)	32 (64·0)	29 (70·7)
August 2021	0	0	0	1 (5·9)	1 (16·7)	0	2	1 (25·0)	1 (12·5)	1 (1·1)	1 (2·0)	0
September 2021	0	0	0	0	0	0	0	0	0	2 (2·2)	1 (2·0)	1 (2·4)

Data shown are n (%) or median (IQR).

[a]Participants with >1 chronic condition are included in each relevant category, and so the percentages for all categories sum to >100 within each column.

[b]As there is no linkage to national testing data, information about prior SARS-CoV-2 infection is reliant on hospital records and patient recall.

COVID-19=coronavirus disease 2019. IQR=interquartile range. SARI=severe acute respiratory infection. SARS-CoV-2=severe acute respiratory syndrome coronavirus 2. TNC=test-negative controls. TPC=test-positive cases.

Table S3: Demographic characteristics of the study population, stratified by SARS-CoV-2 variant periods

Characteristic	Jun 1–Jul 31, 2021 (Alpha, Delta)			Aug 1–Nov 30 2021 (Delta)			Dec 1–31, 2021 (Delta, Omicron clade 21K [BA.1])			Jan 1–Mar 31, 2022 (Mixed Omicron clades 21K [BA.1], 21L [BA.2])			April 1–Sep 5, 2022 (Mixed Omicron clades 21L [BA.2], 22A [BA.4], 22B [BA.5], 22C [BA.2.12.1], 22D [BA.2.75])		
	Total (N=84)	TNC (n=19)	TPC (n=65)	Total (N=221)	TNC (n=75)	TPC (n=146)	Total (N=144)	TNC (n=21)	TPC (n=123)	Total (N=188)	TNC (n=51)	TPC (n=137)	Total (N=69)	TNC (n=29)	TPC (n=40)
**Age at hospitalisation, years**	55·0 (37·8–65·0)	63·0 (57·0–78·5)	50·0 (37·0–59·0)	57·0 (44·0–67·0)	65·0 (55·0–69·0)	51·5 (41·0–63·8)	61·5 (48·8–68·0)	65·0 (54·0–68·0)	61·0 (44·5–68·0)	63·5 (50·8–75·2)	59·0 (45·5–69·0)	66·0 (52·0–77·0)	70·0 (58·0–80·0)	66·0 (49·0–76·0)	72·0 (61·5–80·0)
18–49	35 (41·7)	4 (21·1)	31 (47·7)	80 (36·2)	16 (21·3)	64 (43·8)	41 (28·5)	3 (14·3)	38 (30·9)	45 (23·9)	18 (35·3)	27 (19·7)	13 (18·8)	8 (27·6)	5 (12·5)
50–64	27 (32·1)	6 (31·6)	21 (32·2)	68 (30·8)	21 (28·0)	47 (32·2)	51 (35·4)	6 (28·6)	45 (36·6)	52 (27·7)	15 (29·4)	37 (27·0)	15 (21·7)	6 (20·7)	9 (22·5)
≥65	22 (26·2)	9 (47·4)	13 (20·0)	73 (33·0)	38 (50·7)	35 (24·0)	52 (36·1)	12 (57·1)	40 (32·5)	91 (48·4)	18 (35·3)	73 (53·3)	41 (59·4)	15 (51·7)	26 (65·0)
**Sex**															
Female	36 (42·9)	6 (31·6)	30 (46·2)	94 (42·5)	31 (41·3)	63 (43·2)	60 (41·7)	10 (47·6)	50 (40·7)	74 (39·4)	18 (35·3)	56 (40·9)	33 (47·8)	12 (41·4)	21 (52·5)
Male	48 (57·1)	13 (68·4)	35 (53·8)	127 (57·5)	44 (58·7)	83 (56·8)	84 (58·3)	11 (52·4)	73 (59·3)	114 (60·6)	33 (64·7)	81 (59·1)	36 (52·2)	17 (58·6)	19 (47·5)
**Country**															
Austria	19 (22·6)	0	19 (29·2)	0	0	0	0	0	0	10 (5·3)	0	10 (7·3)	13 (18·8)	2 (6·9)	11 (27·5)
Belgium	14 (16·7)	4 (21·1)	10 (15·4)	92 (41·6)	12 (16·0)	80 (54·8)	21 (14·6)	3 (14·3)	18 (14·6)	15 (8·0)	1 (2·0)	14 (10·2)	1 (1·4)	1 (3·4)	0
Italy	0	0	0	8 (3·6)	0	8 (5·5)	26 (18·1)	1 (4·8)	25 (20·3)	76 (40·4)	27 (52·9)	49 (35·8)	21 (30·4)	8 (27·6)	13 (32·5)
Spain	51 (60·7)	15 (78·9)	36 (55·4)	121 (54·8)	63 (84·0)	58 (49·7)	97 (67·4)	17 (81·0)	80 (65·0)	87 (46·3)	23 (45·1)	64 (46·7)	34 (49·3)	18 (62·1)	16 (40·0)
**Body mass index (BMI) category**															
Underweight (BMI <18·5)	0	0 (0%)	0	2 (0·9)	0	2 (1·4)	0	0	0	3 (1·6)	1 (2·0)	2 (1·5)	0	0	0
Normal/healthy weight (BMI ≥18·5 to <25·0)	8 (9·5)	2 (10·5)	6 (9·2)	23 (10·4)	7 (9·3)	16 (11·0)	12 (8·3)	1 (4·8)	11 (8·9)	50 (26·6)	24 (47·1)	26 (19·0)	28 (40·6)	10 (34·5)	18 (45·0)
Overweight (BMI ≥25·0 to <30·0)	8 (9·5)	1 (5·3)	7 (10·8)	35 (15·8)	7 (9·3)	28 (19·2)	22 (15·3)	4 (19·0)	18 (14·6)	42 (22·3)	13 (25·5)	29 (21·2)	18 (26·1)	8 (27·6)	10 (25·0)
Obese (BMI ≥30·0)	14 (16·7)	2 (10·5)	12 (18·5)	25 (11·3)	6 (8·0)	19 (13·0)	18 (12·5)	1 (4·8)	17 (13·8)	32 (17·0)	6 (11·8)	26 (19·0)	15 (21·7)	9 (31·0)	6 (15·0)
No information	0	0	0	1 (0·5)	1 (1·3)	0	3 (2·1)	0	3 (2·4)	3 (1·6)	0	3 (2·2)	0	0	0
Missing	54 (64·3)	14 (73·7)	40 (61·5)	135 (61·1)	54 (72·0)	81 (55·5)	89 (61·8)	15 (71·4)	74 (60·2)	58 (30·9)	7 (13·7)	51 (37·2)	8 (11·6)	2 (6·9)	6 (15·0)
**Number of chronic conditions**															
0	39 (46·4)	4 (21·1)	35 (53·8)	97 (43·9)	15 (20·0)	82 (56·2)	64 (44·4)	3 (14·3)	61 (49·6)	71 (37·8)	19 (37·3)	52 (38·0)	21 (30·4)	9 (31·0)	12 (30·0)
1	13 (15·5)	4 (21·1)	9 (13·8)	40 (18·1)	16 (21·2)	24 (16·4)	24 (16·7)	3 (14·3)	21 (17·1)	44 (23·4)	12 (23·5)	32 (23·4)	14 (20·3)	6 (20·7)	8 (20·0)
2	15 (17·9)	3 (15·8)	12 (18·5)	43 (19·5)	20 (26·7)	23 (15·8)	35 (24·3)	6 (28·6)	29 (23·6)	34 (18·1)	12 (23·5)	22 (16·1)	16 (23·2)	8 (27·6)	8 (20·0)
≥3	17 (20·2)	8 (42·1)	9 (13·8)	41 (18·6)	24 (32·0)	17 (11·6)	21 (14·6)	9 (42·9)	12 (9·8)	39 (20·7)	8 (15·7)	31 (22·6)	18 (26·1)	6 (20·7)	12 (30·0)
**Participants with chronic condition[a]**															
Asthma	7 (8·3)	2 (10·5)	5 (7·7)	13 (5·9)	8 (10·7)	5 (3·4)	8 (5·6)	4 (19·0)	4 (3·3)	12 (6·4)	5 (9·8)	7 (5·1)	4 (5·8)	2 (6·9)	2 (5·0)
Lung disease	18 (21·4)	9 (47·4)	9 (13·8)	36 (16·3)	22 (29·2)	14 (9·6)	23 (16·0)	9 (42·9)	14 (11·4)	34 (18·1)	13 (25·5)	21 (15·3)	18 (26·1)	9 (31·0)	9 (22·5)
Cardiovascular disease	14 (16·7)	5 (26·3)	9 (13·8)	43 (19·5)	25 (33·3)	18 (12·3)	29 (20·1)	10 (47·6)	19 (15·4)	48 (25·5)	13 (25·5)	35 (25·5)	25 (36·2)	9 (31·0)	16 (40·0)
Hypertension	25 (29·8)	7 (36·8)	18 (27·7)	60 (27·1)	28 (37·3)	32 (21·9)	44 (30·6)	9 (42·9)	35 (28·5)	65 (34·6)	12 (23·5)	53 (38·7)	33 (47·8)	12 (41·4)	21 (52·5)
Chronic liver disease	4 (4·8)	1 (5·3)	3 (4·6)	12 (5·4)	7 (9·2)	5 (3·4)	3 (2·1)	0	3 (2·4)	10 (5·3)	1 (2·0)	9 (6·6)	2 (2·9)	0	2 (5·0)
Chronic kidney disease	12 (14·3)	6 (31·6)	6 (9·2)	18 (8·1)	12 (16·0)	6 (4·1)	7 (4·9)	2 (9·5)	5 (4·1)	23 (12·2)	4 (7·8)	19 (13·9)	3 (4·3)	2 (6·9)	1 (2·5)
Type 2 diabetes	12 (14·3)	4 (21·1)	8 (12·3)	43 (19·5)	15 (20·0)	28 (19·2)	19 (13·2)	3 (14·3)	16 (13·0)	34 (18·1)	4 (7·8)	30 (21·9)	10 (14·5)	2 (6·9)	8 (20·0)
Cancer	8 (9·5)	3 (15·8)	5 (7·7)	26 (11·8)	14 (18·7)	12 (8·2)	12 (8·3)	6 (28·6)	6 (4·9)	19 (10·1)	8 (15·7)	11 (8·0)	6 (8·7)	3 (10·3)	3 (7·5)
Immunodeficiency (or organ transplant)	4 (4·8)	1 (5·3)	3 (4·6)	27 (12·2)	14 (18·7)	13 (8·9)	9 (6·3)	3 (14·3)	6 (4·9)	7 (3·7)	3 (5·9)	4 (2·9)	5 (7·2)	3 (10·3)	2 (5·0)

Characteristic	Jun 1–Jul 31, 2021 (Alpha, Delta) Total (N=84)	TNC (n=19)	TPC (n=65)	Aug 1–Nov 30 2021 (Delta) Total (N=221)	TNC (n=75)	TPC (n=146)	Dec 1–31, 2021 (Delta, Omicron clade 21K [BA.1]) Total (N=144)	TNC (n=21)	TPC (n=123)	Jan 1–Mar 31, 2022 (Mixed Omicron clades 21K [BA.1], 21L [BA.2]) Total (N=188)	TNC (n=51)	TPC (n=137)	April 1–Sep 5, 2022 (Mixed Omicron clades 21L [BA.2], 22A [BA.4], 22B [BA.5], 22C [BA.2.12.1], 22D [BA.2.75]) Total (N=69)	TNC (n=29)	TPC (n=40)
**Pregnancy**	n=36	n=6	n=30	n=94	n=31	n=63	n=60	n=10	n=50	n=74	n=18	n=56	n=33	n=12	n=21
No	33 (91·7)	6 (100)	27 (90·0)	87 (92·6)	30 (96·8)	57 (90·5)	55 (91·7)	10 (100)	45 (90·0)	65 (87·8)	17 (94·4)	48 (85·7)	31 (93·9)	12 (100)	19 (90·5)
Yes	1 (2·8)	0	1 (3·3)	7 (7·4)	1 (3·2)	6 (9·5)	4 (6·7)	0	4 (8·0)	6 (8·1)	0	6 (10·7)	2 (6·1)	0	2 (9·5)
No information	0	0	0	0	0	0	0	0	0	1 (1·4)	1 (5·6)	0	0	0	0
Missing	2 (5·6)	0	2 (6·7)	0	0	0	1 (1·7)	0	1 (2·0)	2 (2·7)	0	2 (3·6)	0	0	0
**Prior SARS-CoV-2 infection[b]**															
No prior infection	81 (96·4)	16 (84·2)	65 (100)	145 (65·6)	48 (64·0)	97 (66·4)	76 (52·8)	9 (42·9)	67 (54·5)	115 (61·2)	31 (60·8)	84 (61·3)	32 (46·4)	11 (37·9)	21 (52·5)
Yes – clinical diagnosis	0	0	0	0	0	0	1 (0·7)	0	1 (0·8)	2 (1·1)	1 (2·0)	1 (0·7)	0	0	0
Yes – laboratory-confirmed	3 (3·6)	3 (15·8)	0	5 (2·3)	3 (4·0)	2 (1·4)	2 (1·4)	2 (9·5)	0	5 (2·7)	3 (5·9)	2 (1·5)	4 (5·8)	2 (6·9)	2 (5·0)
No information	0	0	0	0	0	0	0	0	0	1 (0·5)	0	1 (0·7)	1 (1·4)	0	1 (2·5)
Missing	0	0	0	71 (32·1)	24 (32·0)	47 (32·2)	65 (45·1)	10 (47·6)	55 (44·7)	65 (34·6)	16 (31·4)	49 (35·8)	32 (46·4)	16 (55·2)	16 (40·0)
**Vaccinated for influenza within 12 months prior to SARI hospital admission**															
No	51 (60·7)	13 (68·4)	38 (58·5)	110 (49·8)	38 (50·7)	72 (49·3)	98 (68·1)	15 (71·4)	83 (67·5)	153 (81·4)	48 (94·1)	105 (76·6)	58 (84·1)	26 (89·7)	32 (80·0)
Yes	10 (11·9)	6 (31·6)	4 (6·2)	30 (13·6)	19 (25·2)	11 (7·5)	16 (11·1)	5 (23·8)	11 (8·9)	9 (4·8)	3 (5·9)	6 (4·4)	7 (10·1)	2 (6·9)	5 (12·5)
No information	23 (27·4)	0	23 (35·4)	76 (34·4)	16 (21·3)	60 (41·1)	23 (16·0)	1 (4·8)	22 (17·9)	19 (10·1)	0	19 (13·9)	4 (5·8)	1 (3·4)	3 (7·5)
Missing	0	0	0	5 (2·3)	2 (2·7)	3 (2·1)	7 (4·9)	0	7 (5·7)	7 (3·7)	0	7 (5·1)	0	0	0
**Received any pneumococcal vaccine**															
No	49 (58·3)	12 (63·2)	37 (56·9)	112 (50·7)	35 (46·7)	77 (52·7)	105 (72·9)	16 (76·2)	89 (72·4)	147 (78·2)	46 (90·2)	101 (73·7)	55 (79·7)	26 (89·7)	29 (72·5)
Yes	12 (14·3)	7 (36·8)	5 (7·7)	29 (13·1)	22 (29·3)	7 (4·8)	6 (4·2)	4 (19·0)	2 (1·6)	14 (7·4)	4 (7·8)	10 (7·3)	5 (7·2)	2 (6·9)	3 (7·5)
No information	23 (27·4)	0	23 (35·4)	75 (33·9)	16 (21·3)	59 (40·4)	26 (18·1)	1 (4·8)	25 (20·3)	21 (11·2)	1 (2·0)	20 (14·6)	9 (13·0)	1 (3·4)	8 (20·0)
Missing	0	0	0	5 (2·3)	2 (2·7)	3 (2·1)	7 (4·9)	0	7 (5·7)	6 (3·2)	0	6 (4·4)	0	0	0
**Smoking history**															
Never smoked	19 (22·6)	4 (21·1)	15 (23·1)	71 (32·1)	23 (30·7)	48 (32·9)	65 (45·1)	5 (23·8)	60 (48·8)	65 (34·6)	11 (21·6)	54 (39·4)	22 (31·9)	3 (10·3)	19 (47·5)
Ex-smoker	12 (14·3)	5 (26·3)	7 (10·8)	36 (16·3)	15 (20·0)	21 (14·4)	37 (25·7)	8 (38·1)	29 (23·6)	39 (20·7)	8 (15·7)	31 (22·6)	21 (30·4)	12 (41·4)	9 (22·5)
Occasional smoker	0	0	0	5 (2·3)	2 (2·7)	3 (2·1)	1 (0·7)	1 (4·8)	0	4 (2·1)	2 (3·9)	2 (1·5)	1 (1·4)	1 (3·4)	0
Daily smoker	11 (13·1)	5 (26·3)	6 (9·2)	26 (11·8)	20 (26·7)	6 (4·1)	13 (9·0)	7 (33·3)	6 (4·9)	15 (8·0)	6 (11·8)	9 (6·6)	19 (27·5)	9 (31·0)	10 (25·0)
No information	42 (50·0)	5 (26·3)	37 (56·9)	76 (34·4)	11 (14·7)	65 (44·5)	27 (18·8)	0	27 (22·0)	64 (34·0)	24 (47·1)	40 (29·2)	6 (8·7)	4 (13·8)	2 (5·0)
Missing	0	0	0	7 (3·2)	4 (5·3)	3 (2·1)	1 (0·7)	0	1 (0·8)	1 (0·5)	0	1 (0·7)	0	0	0
**Long-term care facility resident**															
No	78 (92·9)	16 (84·2)	62 (95·4)	209 (94·6)	68 (90·7)	141 (96·6)	142 (98·6)	21 (100)	121 (98·4)	185 (98·4)	51 (100)	134 (97·8)	68 (98·6)	29 (100)	39 (97·5)
Yes	1 (1·2)	0	1 (1·5)	1 (0·5)	1 (1·3)	0	0	0	0	2 (1·1)	0	2 (1·5)	0	0	0
No information	5 (6·0)	3 (15·8)	2 (3·1)	4 (1·8)	2 (2·7)	2 (1·4)	1 (0·7)	0	1 (0·8)	0	0	0	1 (1·4)	0	1 (2·5)
Missing	0	0	0	7 (3·2)	4 (5·3)	3 (2·1)	1 (0·7)	0	1 (0·8)	1 (0·5)	0	1 (0·7)	0	0	0
**Healthcare worker**															
No	79 (94·0)	19 (100)	60 (92·3)	153 (69·2)	56 (74·7)	97 (66·4)	110 (76·4)	17 (81·0)	93 (75·6)	150 (79·8)	37 (72·5)	113 (82·5)	43 (62·3)	14 (48·3)	29 (72·5)
Yes	0	0	0	0	0	0	0	0	0	0	0	0	1 (1·4)	0	1 (2·5)

Characteristic	Jun 1–Jul 31, 2021 (Alpha, Delta)			Aug 1–Nov 30 2021 (Delta)			Dec 1–31, 2021 (Delta, Omicron clade 21K [BA.1])			Jan 1–Mar 31, 2022 (Mixed Omicron clades 21K [BA.1], 21L [BA.2])			April 1–Sep 5, 2022 (Mixed Omicron clades 21L [BA.2], 22A [BA.4], 22B [BA.5], 22C [BA.2.12.1], 22D [BA.2.75])		
	**Total (N=84)**	**TNC (n=19)**	**TPC (n=65)**	**Total (N=221)**	**TNC (n=75)**	**TPC (n=146)**	**Total (N=144)**	**TNC (n=21)**	**TPC (n=123)**	**Total (N=188)**	**TNC (n=51)**	**TPC (n=137)**	**Total (N=69)**	**TNC (n=29)**	**TPC (n=40)**
No information	5 (6·0)	0	5 (7·7)	60 (27·1)	15 (20·0)	45 (30·8)	34 (23·6)	4 (19·0)	30 (24·4)	37 (19·7)	14 (27·5)	23 (16·8)	25 (36·2)	15 (51·7)	10 (25·0)
Missing	0	0	0	8 (3·6)	4 (5·3)	4 (2·7)	0	0	0	1 (0·5)	0	1 (0·7)	0	0	0
**Genetic variant**	n=65	n/a	n=65	n=146	n/a	n=146	n=123	n/a	n=123	n=137	n/a	n=137	n=40	n/a	n=40
Alpha	0	0	0	0	0	0	0	0	0	0	0	0	0	0	0
Beta	0	0	0	0	0	0	0	0	0	0	0	0	0	0	0
Delta	13 (20·0)	0	13 (20·0)	43 (29·5)	0	43 (29·5)	53 (43·1)	0	53 (43·1)	12 (8·8)	0	12 (8·8)	0	0	0
Omicron	0	0	0	0	0	0	6 (4·9)	0	6 (4·9)	67 (48·9)	0	67 (48·9)	13 (32·5)	0	13 (32·5)
Not interpretable/unknown	0	0	0	1 (0·7)	0	1 (0·7)	0	0	0	1 (0·7)	0	1 (0·7)	0	0	0
Missing	52 (80·0)	0	52 (80·0)	102 (69·9)	0	102 (69·9)	64 (52·0)	0	64 (52·0)	57 (41·6)	0	57 (41·6)	27 (67·5)	0	27 (67·5)
**Vaccinated against COVID-19**															
No	79 (94·0)	15 (78·9)	64 (98·5)	160 (72·4)	38 (50·7)	122 (83·6)	109 (75·7)	10 (47·6)	99 (80·5)	172 (91·5)	46 (90·2)	126 (92·0)	66 (95·7)	26 (89·7)	40 (100)
Fully vaccinated with two doses of AZD1222	5 (6·0)	4 (21·1)	1 (1·5)	61 (27·6)	37 (49·3)	24 (16·4)	35 (24·3)	11 (52·4)	24 (19·5)	16 (8·5)	5 (9·8)	11 (8·0)	3 (4·3)	3 (10·3)	0
**Month and year of second AZD1222 dose**	n=5	n=4	n=1	n=61	n=37	n=24	n=35	n=11	n=24	n=16	n=5	n=11	n=3	n=3	n=0
March 2021	0	0	0	1 (1·6)	0	1 (4·2)	0	0	0	0	0	0	0	0	0
April 2021	0	0	0	0	0	0	0	0	0	0	0	0	0	0	0
May 2021	0	0	0	3 (4·9)	1 (2·7)	2 (8·3)	0	0	0	2 (12·5)	2 (40·0)	0	0	0	0
June 2021	5 (100)	4 (100)	1 (100)	18 (29·5)	12 (32·4)	6 (25·0)	8 (22·9)	2 (18·2)	6 (25·0)	1 (6·3)	0	1 (9·1)	1 (33·3)	1 (33·3)	0
July 2021	0	0	0	38 (62·3)	23 (62·2)	15 (62·5)	25 (71·4)	8 (72·7)	17 (70·8)	12 (75·0)	3 (60·0)	9 (81·8)	0	0	0
August 2021	0	0	0	0	0	0	1 (2·9)	1 (9·1)	0	1 (6·3)	0	1 (9·1)	2 (66·7)	2 (66·7)	0
September 2021	0	0	0	1 (1·6)	1 (2·7)	0	1 (2·9)	0	1 (4·2)	0	0	0	0	0	0

Data shown are n (%) or median (IQR).

[a]Participants may be included in multiple categories, so percentages do not sum to 100.

[b]As there is no linkage to national testing data, information about prior SARS-CoV-2 infection is reliant on hospital records and patient recall.

COVID-19=coronavirus disease 2019. IQR=interquartile range. SARI=severe acute respiratory infection. SARS-CoV-2=severe acute respiratory syndrome coronavirus 2. TNC=test-negative controls. TPC=test-positive cases.

***Table S4*: Cumulative symptom onset date-adjusted VE estimates by time since last dose and by SARS-CoV-2 genetic variant in SARI patients who had received two doses of AZD1222**

Time since last dose of AZD1222	Symptom onset-date-adjusted VE (95% CI)					
		Variant				
	**All**	**Alpha**	**Beta**	**Delta**	**Omicron**	**Missing**
**Time period**						
2 to ≤8 weeks	91·3 (35·5–98·8)	NE	NE	85·0 (-96·3–98·8)	NE	96·3 (53·5–99·7)
>8 to ≤16 weeks	79·2 (42·7–92·5)	NE	NE	73·5 (-8·7–93·5)	NE	85·2 (50·9–95·6)
>16 to ≤24 weeks	70·5 (39·9–85·5)	NE	NE	60·9 (-5·3–85·5)	80·4 (-399·3–99·2)	69·1 (33·4–85·7)
>24 to ≤32 weeks	54·5 (-53·3–86·5)	NE	NE	90·0 (-20·2–99·2)	-40·2 (-604·4–72·1)	86·3 (8·1–98·0)
>32 weeks	80·9 (-7·1–96·6)	NE	NE	NE	69·7 (-195·5–96·9)	79·0 (-93·0–97·7)
**Cumulative time**						
≤8 weeks	91·3 (35·5–98·8)	NE	NE	85·0 (-96·3–98·8)	NE	96·3 (53·5–99·7)
≤16 weeks	82·7 (57·1–93·0)	NE	NE	77·0 (21·7–93·2)	NE	89·0 (66·7–96·4)
≤24 weeks	75·5 (56·7–86·1)	NE	NE	67·3 (30·1–84·7)	80·4 (-399·4–99·2)	77·9 (58·2–88·3)
≤32 weeks	72·3 (53·4–83·6)	NE	NE	69·0 (35·0–85·2)	-17·3 (-437·5–74·4)	78·7 (60·7–88·4)
**All**	73·1 (55·8–83·7)	NE	NE	69·3 (35·7–85·3)	27·2 (-136·5–77·6)	78·8 (61·7–88·3)

CI=confidence interval. NE=not estimable. VE=vaccine effectiveness.

Table S5: **Confounder-adjusted VE estimates by calendar time-period in SARI patients who had received two doses of AZD1222 and were hospitalised between June 1st, 2021, and September 5th, 2022.** The calendar time-intervals coincide with changes in the dominance of SARS-CoV-2 genetic variants as reported by the European Centre for Disease Prevention and Control (https://www.ecdc.europa.eu/en/covid-19/situation-updates/variants-dashboard) and CoVariants: SARS-CoV-2 Mutations and Variants of Interest (covariants.org).

Calendar time-period	Exposure	Mean time since last dose of AZD1222, days (range)	Test-negative controls	Test-positive cases	Symptom-onset adjusted VE, % (95% CI)	Confounder-adjusted VE, % (95% CI)
Jun 1–Jul 31, 2021	Vaccinated	29·0 (14–45)	4	1	94·7 (34·4–99·6)	91·0 (-20·5–99·3)
(Alpha, Delta)	Unvaccinated	NA	15	64		
Aug 1–Nov 30 2021	Vaccinated	115·7 (42–196)	37	24	69·9 (32·7–86·5)	64·6 (6·1–86·6)
(Delta)	Unvaccinated	NA	38	122		
Dec 1–31, 2021	Vaccinated	156·0 (107–186)	11	24	69·8 (10·3–89·8)	62·6 (-51·7–90·8)
(Delta, Omicron clade 21K [BA.1])	Unvaccinated	NA	10	99		
Jan 1–Mar 31, 2022	Vaccinated	210·2 (157–264)	5	11	23·2 (-184·3–79·3)	23·1 (-216·5–81·3)
(Mixed Omicron clades 21K [BA.1] and 21L [BA.2])	Unvaccinated	NA	46	126		
April 1–Sep 5, 2022	Vaccinated	319·7 (296–348)	3	0	NE	NE
(Mixed Omicron clades 21L [BA.2], 22A [BA.4], 22B [BA.5], 22C [BA.2.12.1], 22D [BA.2.75])	Unvaccinated	NA	26	40		

CI=confidence interval. VE=vaccine effectiveness. NA=not applicable. NE=not estimable. SARI=severe acute respiratory infection.

Figure S1*: Attrition diagram of participants in the COVIDRIVE study and the SARI patients in this study of AZD1222*.

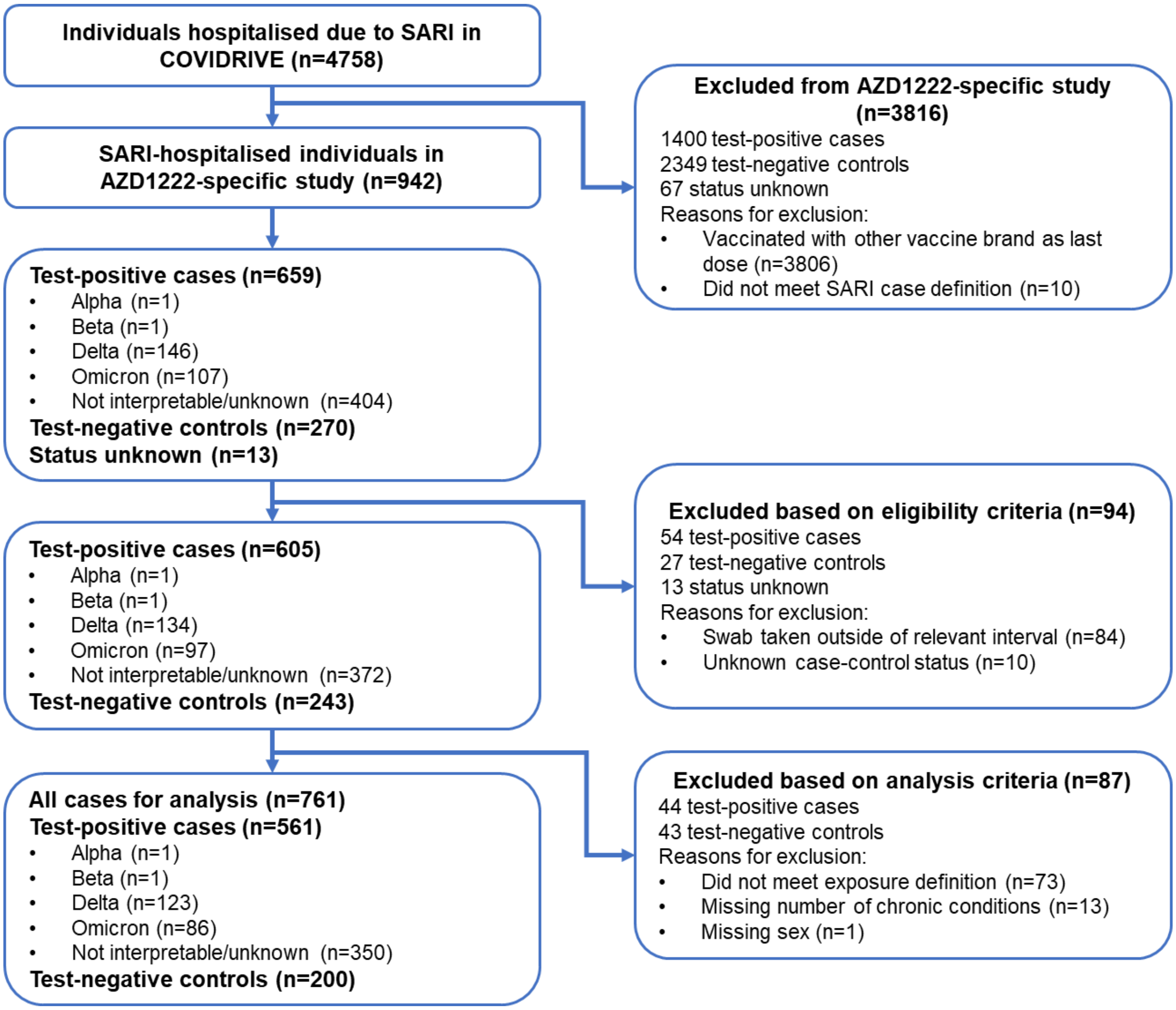

SARI=severe acute respiratory infection.
